# *Solanum* pan-genetics reveals paralogues as contingencies in crop engineering

**DOI:** 10.1038/s41586-025-08619-6

**Published:** 2025-03-05

**Authors:** Matthias Benoit, Katharine M. Jenike, James W. Satterlee, Srividya Ramakrishnan, Iacopo Gentile, Anat Hendelman, Michael J. Passalacqua, Hamsini Suresh, Hagai Shohat, Gina M. Robitaille, Blaine Fitzgerald, Michael Alonge, Xingang Wang, Ryan Santos, Jia He, Shujun Ou, Hezi Golan, Yumi Green, Kerry Swartwood, Nicholas G. Karavolias, Gina P. Sierra, Andres Orejuela, Federico Roda, Sara Goodwin, W. Richard McCombie, Elizabeth B. Kizito, Edeline Gagnon, Sandra Knapp, Tiina E. Särkinen, Amy Frary, Jesse Gillis, Joyce Van Eck, Michael C. Schatz, Zachary B. Lippman

**Affiliations:** 1 Howard Hughes Medical Institute, Cold Spring Harbor Laboratory, Cold Spring Harbor, NY USA; 2 Department of Genetic Medicine, Johns Hopkins School of Medicine, Baltimore, MD USA; 3 Department of Computer Science, Johns Hopkins University, Baltimore, MD USA; 4 Cold Spring Harbor Laboratory, Cold Spring Harbor, NY USA; 5 School of Biological Sciences, Cold Spring Harbor Laboratory, Cold Spring Harbor, NY USA; 6 SiteKicks.ai, Setauket, NY USA; 7 Boyce Thompson Institute, Ithaca, NY USA; 8 Max Planck Tandem Group, Facultad de Ciencias, Universidad Nacional de Colombia, Bogotá, Colombia; 9 Departamento de Biología, Facultad de Ciencias Exactas y Naturales, Universidad de Cartagena, Cartagena de Indias, Colombia; 10 Faculty of Agricultural Sciences, Uganda Christian University, Mukono, Uganda; 11 Department of Integrative Biology, University of Guelph, Guelph, Ontario Canada; 12 Royal Botanic Garden Edinburgh, Edinburgh, UK; 13 Natural History Museum, London, UK; 14 Department of Biological Sciences, Mount Holyoke College, South Hadley, MA USA; 15 Physiology Department and Donnelly Centre for Cellular and Biomolecular Research, University of Toronto, Toronto, Ontario Canada; 16 Plant Breeding and Genetics Section, School of Integrative Plant Science, Cornell University, Ithaca, NY USA; 17 Present Address: LIPME, Université de Toulouse, INRAE, CNRS, Castanet-Tolosan, France; 18 Present Address: Ohalo Genetics, Aptos, CA USA; 19 Present Address: Verve Therapeutics, Boston, MA USA; 20 Present Address: Department of Molecular Genetics, Ohio State University, Columbus, OH USA; 21 Present Address: School of Life Sciences, Technical University of Munich, Freising, Germany

**Keywords:** Plant domestication, Agricultural genetics, Genome informatics, Evolutionary genetics

## Abstract

Pan-genomics and genome-editing technologies are revolutionizing breeding of global crops^1,2^. A transformative opportunity lies in exchanging genotype-to-phenotype knowledge between major crops (that is, those cultivated globally) and indigenous crops (that is, those locally cultivated within a circumscribed area)^3–5^ to enhance our food system. However, species-specific genetic variants and their interactions with desirable natural or engineered mutations pose barriers to achieving predictable phenotypic effects, even between related crops^6,7^. Here, by establishing a pan-genome of the crop-rich genus *Solanum*^8^ and integrating functional genomics and pan-genetics, we show that gene duplication and subsequent paralogue diversification are major obstacles to genotype-to-phenotype predictability. Despite broad conservation of gene macrosynteny among chromosome-scale references for 22 species, including 13 indigenous crops, thousands of gene duplications, particularly within key domestication gene families, exhibited dynamic trajectories in sequence, expression and function. By augmenting our pan-genome with African eggplant cultivars^9^ and applying quantitative genetics and genome editing, we dissected an intricate history of paralogue evolution affecting fruit size. The loss of a redundant paralogue of the classical fruit size regulator *CLAVATA3* (*CLV3*)^10,11^ was compensated by a lineage-specific tandem duplication. Subsequent pseudogenization of the derived copy, followed by a large cultivar-specific deletion, created a single fused *CLV3* allele that modulates fruit organ number alongside an enzymatic gene controlling the same trait. Our findings demonstrate that paralogue diversifications over short timescales are underexplored contingencies in trait evolvability. Exposing and navigating these contingencies is crucial for translating genotype-to-phenotype relationships across species.

## Abstract (web summary)

Gene duplication and subsequent paralogue diversification are major obstacles to genotype-to-phenotype predictability.

## Main

Global food production is based on a small number of intensively bred commodity crops from three plant families^12^: grasses (corn, rice, wheat), legumes (soybean) and nightshades (potato, tomato). By contrast, indigenous crops comprise a heterogeneous group of hundreds of species that could contribute to agricultural biodiversity and resilience^3^. Many indigenous crops belong to the same families as major crops but are differentiated by their limited cultivation range and scale of production^5^. For example, the grasses finger millet (*Eleusine coracana*) and teff (*Eragrostis tef*), as well as the legumes cowpea (*Vigna unguiculata*) and pigeonpea (*Cajanus cajan*), are locally adapted and important to diets in regions of Africa and Asia^13–15^. Within the nightshade (Solanaceae) family, the genus *Solanum* contains dozens of crops and many edible wild species across specific regions of Africa and South America, consumed for their leaves and/or fruits. Prominent among these are African eggplant (*Solanum* *aethiopicum*), naranjilla (*Solanum* *quitoense*) and pepino (*Solanum* *muricatum*)^16,17^.

Indigenous crops are viewed through different lenses—agricultural, ethnobotanical and scientific—each with its own unique biases and objectives^3–5,18^. Bridging and harmonizing these viewpoints offers an opportunity to better serve local communities and encourage broader adoption. Breeding of indigenous crops has been limited, and it is assumed that decades of research on major crops, along with advances in genome-sequencing and genome-editing technologies, can help to address undesirable ancestral traits that limit productivity^19^. Engineering beneficial mutations in these crops could expand our current genetically narrow, industrialized agricultural systems^3,20^. Despite progress in genome engineering, background dependencies—species-specific genetic modifiers that can cause unpredictable phenotypic outcomes, even between closely related varieties—remain underappreciated barriers^21^. Plant breeders have long lamented that beneficial alleles and quantitative trait loci (QTLs) often underperform when transferred to different backgrounds owing to interactions among variants^22,23^, a challenge that will persist with genome editing^24,25^.

Our tomato pan-genome and associated functional genetics demonstrated that gene duplications can be potent sources of background modifiers^26,27^. Duplications result in genetic redundancy, which permits mutations to accumulate in coding and *cis*-regulatory sequences through genetic drift. Consequently, paralogue redundancy can degrade, leading to three outcomes over long evolutionary time: gene loss (pseudogenization), partitioning of ancestral functions (subfunctionalization) or gain of new functions (neofunctionalization)^28,29^. Less is known about how paralogues diverge over shorter timescales, although interactions between paralogues underlie notable examples of emergence and suppression of genetic incompatibilities^30,31^. Genomic and functional studies of paralogues and their interactions have primarily focused on comparisons within species or between widely diverged lineages, leaving intermediate changes in sequence, expression and function largely unexplored. A deeper understanding of paralogue histories and their interdependencies could improve our ability to predict phenotypic outcomes when applying genetic knowledge across closely related species.

Here we present a *Solanum* pan-genome and use it alongside pan-genetics—comparative forward and reverse genetics across related species—to analyse paralogue evolutionary dynamics in depth. We demonstrate the value of resolving these previously underexplored contingencies as we strive to improve indigenous crops for both local and broader climate-resilient agriculture.

## A pan-genome of the genus *Solanum*

**Fig. 1:**
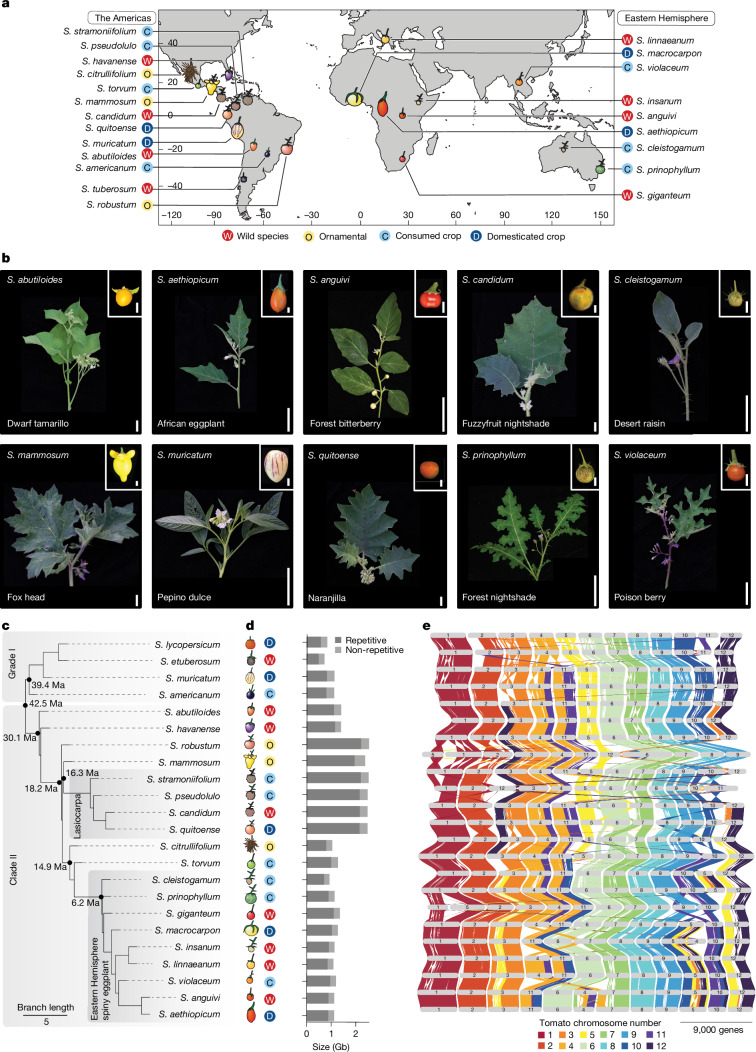
The *Solanum* pan-genome captures the phenotypic, ecological, agricultural and genomic diversity of this crop-rich genus. **a**, Approximate centroid of the native range for the 22 selected *Solanum* species, grouped by type of agricultural use: wild (W), locally important and consumed (C), ornamental (O) and domesticated (D). **b**, The phenotypic diversity of shoots and fruits from a subset of *Solanum* species in the pan-genome. Scale bars, 5 cm (shoots) and 1 cm (fruits). **c**, Orthogroup-based phylogeny of the *Solanum* pan-genome recapitulates the major clades, grade I and clade II. The branch lengths reflect coalescent units. Ma, million years ago. **d**, Genome size (Gb) and representation of non-repetitive (light grey) and repetitive (dark grey) sequences of each species of the *Solanum* pan-genome. **e**, GENESPACE plot showing gene macrosynteny across the pan-genome relative to tomato. Scale bar, 9,000 genes.

With its extensive genomic and genetic tools^32,33^, *Solanum* is a leading system to study paralogue evolution. The genus is one of the most species-rich, ecologically diverse and economically important plant genera^16,17^. It spans approximately 6–43 million years of evolution^8,34^ and includes the major crops eggplant (*Solanum melongena*), potato (*Solanum tuberosum*) and tomato (*Solanum lycopersicum*), and at least 20 indigenous crops such as African eggplant (*S. aethiopicum*), naranjilla (*S. quitoense*) and pepino (*S. muricatum*)^35^. We selected 22 species encompassing a broad phylogenetic sample of the ecological (Fig. 1a), phenotypic (Fig. 1b and Supplementary Fig. 1) and taxonomic (Fig. 1c and Supplementary Table 1) diversity within *Solanum*, including regionally important indigenous crops and ornamental species and several of their wild progenitors. These species are grouped into four main categories that reflect the spectrum of plant use and domestication: wild (W), locally important and consumed (C), ornamental (O) and domesticated food crop (D) (Fig. 1a,b). Using PacBio HiFi sequencing and other long-range scaffolding data, we assembled chromosome-scale genomes for all 22 species, including phased haplotypes of the clonally propagated and highly heterozygous pepino, for a total of 23 assemblies reaching reference quality (average quality value (QV) > 53; average post-contamination screened contig N50 (average weighted contig length) = 66.7 Mb; average benchmarking universal single copy orthologues (BUSCO), 96.9%) (Supplementary Fig. 2a,b and Supplementary Table 2). Final genome sizes ranged from around 713 Mb (*Solanum etuberosum*) to about 2.5 Gb (*Solanum robustum*), with members of the *Lasiocarpa* subclade having four out of the five largest genomes (Fig. 1d). An integrated gene prediction strategy for annotation based on liftover from community-established reference genomes of tomato (Heinz) and eggplant (Brinjal) along with de novo gene model calling using species-specific multi-tissue RNA-sequencing (RNA-seq) enabled us to identify 825,493 gene models across the pan-genome (Supplementary Fig. 2c, Supplementary Table 3 and Methods). Of these, 495,429 (about 60%) were shared across all samples as revealed by shared orthology (core genes), demonstrating these species’ relatively close evolutionary relationships.

An orthologue-based phylogenetic tree divided the 22 species into two major clades, consistent with previous studies^34,35^. Using existing nomenclature^35^, grade I (previously clade I, but redefined as grade I owing to a set of paraphyletic clades that do not form a monophyletic group) included the major crops tomato and potato, whereas clade II contained all prickly species^32,33^, including the three cultivated eggplant species: *S. melongena* (Brinjal eggplant), *S. aethiopicum* (African eggplant) and * Solanum macrocarpon* (Gboma eggplant) (Fig. 1c). Consistent with other plant pan-genomes^36,37^, although gene content was largely uniform, species-specific increases in repetitive content driven primarily by a rapid expansion of retrotransposon families correlated strongly with genome size expansion (Fig. 1d, Supplementary Tables 2 and 4 and Supplementary Fig. 3a). We used a *k*-mer analysis to assess the genomic diversity within each species relative to the rest of the pan-genome. The pan-genomic *k*-mer content varied by clade, with 11 species containing more than 25% species-specific sequences (Supplementary Fig. 3a,b). We observed broad conservation of gene macrosynteny throughout the pan-genome, with the highest conservation on chromosomes 1, 2, 6 and 9 (Fig. 1e). This analysis also revealed large structural rearrangements across the genus, particularly within subclades of clade II, including megabase-scale inversions and translocations involving chromosomes 3, 5, 10 and 12 (Fig. 1e). These high-quality genomes provided a foundation for capturing genetic diversity across the *Solanum* from the clade to the species level, setting the stage for an analysis of paralogue evolutionary dynamics and their effects on genotype-to-phenotype relationships.

**Extended Data Fig. 1:**
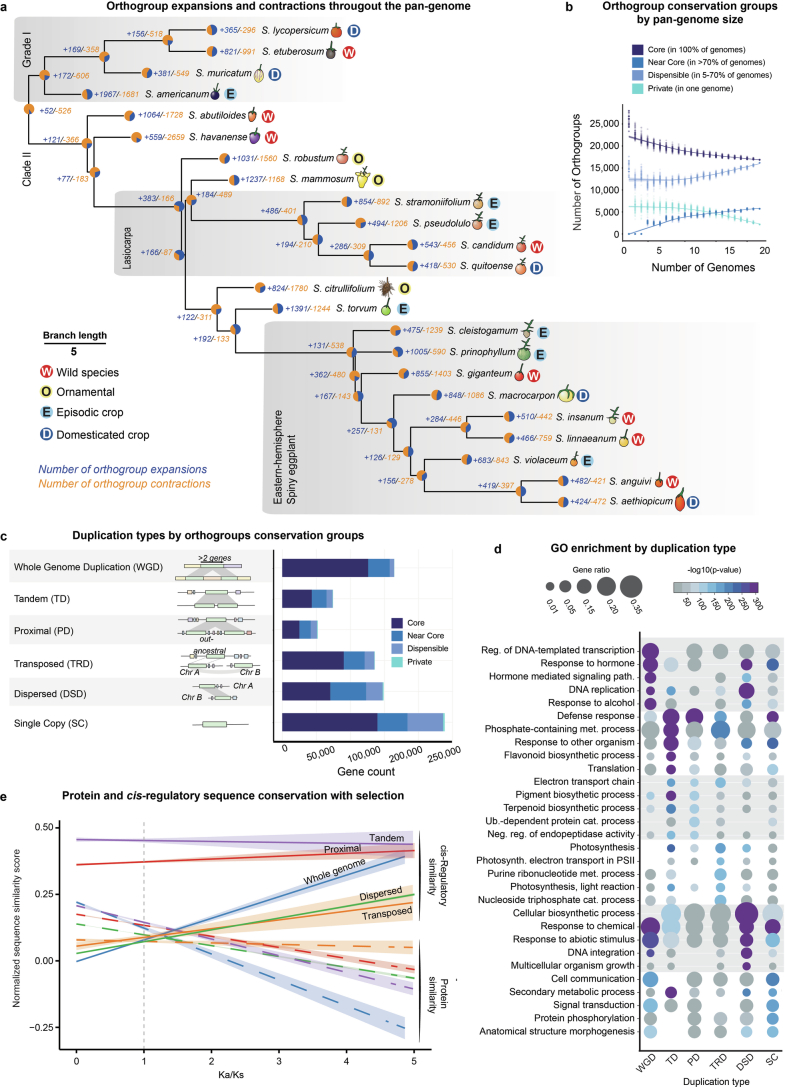
Pan-genomic analysis of orthogroup conservation and diversity of gene duplications. **(a)** Orthogroups expansions and contractions across the pan-genome. The orthogroup-based phylogeny is adapted from Fig. 1c. The estimated expansion (blue) and contraction (orange) rates of orthogroups are shown at each node. **(b)** Cumulative curves showing detection of the four orthogroup conservation groups as a function of the number of species available in the pan-genome. **(c)** Schematic of the potential mechanisms underlying different gene duplication categories, also showing non-duplicated single copy genes for context (left). Stacked bar chart showing the number of genes derived from the different types of duplication sorted by orthogroup conservation groups (right). WGD: whole-genome duplication; TD: tandem duplication; PD: proximal duplication; TRD: transposed duplication; DSD: dispersed duplication; SC: single copy. **(d)** Functional enrichment of gene duplication types detected across the pan-genome. The top five enriched GO terms per duplication type are shown. Gene ratio represents the number of genes with a specific GO term divided by the total number of genes with GO terms in that category. **(e)** Divergence of protein and *cis*-regulatory sequences across increasing evolutionary pressure, as measured by Ka/Ks values, for the indicated types of gene duplication. BLASTP (protein sequence conservation) and LastZ (*cis*-regulatory sequence conservation from the Conservatory algorithm) normalized alignment scores were used to plot the predicted mean and 95% confidence interval (see Supplementary Table 5 for statistical analysis).

To develop a comprehensive view of paralogue evolutionary dynamics across *Solanum*, we first reconstructed the genus-wide history of orthogroup expansion and contraction events from gene families across the 22 species (Extended Data Fig. 1a, Supplementary Fig. 4a and Supplementary Results). We classified orthogroups on the basis of their representation in the pan-genome, as core (present in 100% of the genomes), near core (present in >70% of genomes), dispensable (present in 5–70% of genomes) and private (found in one genome only) (Extended Data Fig. 1b and Supplementary Results). Across all orthogroups, gene duplications were widespread and functionally diverse, with 575,464 duplicates identified across the pan-genome (Extended Data Fig. 1c,d and Supplementary Results). We classified the duplications on the basis of their genomic context as whole-genome duplications (WGD) or single-gene duplications, including tandem, proximal, transposed or dispersed^38^, and assessed their functional enrichment (Extended Data Fig. 1c,d and Supplementary Results). We next compared coding and regulatory sequence evolution across the duplication types (Extended Data Fig. 1e, Supplementary Fig. 4b–e and Supplementary Results). As might be expected, tandem and proximal duplicates, which typically originate from relatively recent structural changes, consistently show high levels of *cis*-regulatory conservation, regardless of selection on protein sequence. By contrast, the other three classes—WGD, dispersed and transposed—show a trend of greater *cis*-regulatory sequence conservation as coding sequence divergence progresses. This finding, although counterintuitive under the assumption that high protein divergence suggests subfunctionalization or neofunctionalization, implies that expression patterns in many paralogue pairs may remain more closely conserved among non-locally duplicated, ancient paralogues. This conservation occurs even as their protein sequences diversify, although not necessarily in function. Broader and deeper sampling of tissues and expression profiles, including single-cell RNA-seq, could reveal specific evolutionary trends in the relationship between *cis*-regulatory and protein changes.

## Transcriptomic fates of retained paralogues

**Fig. 2:**
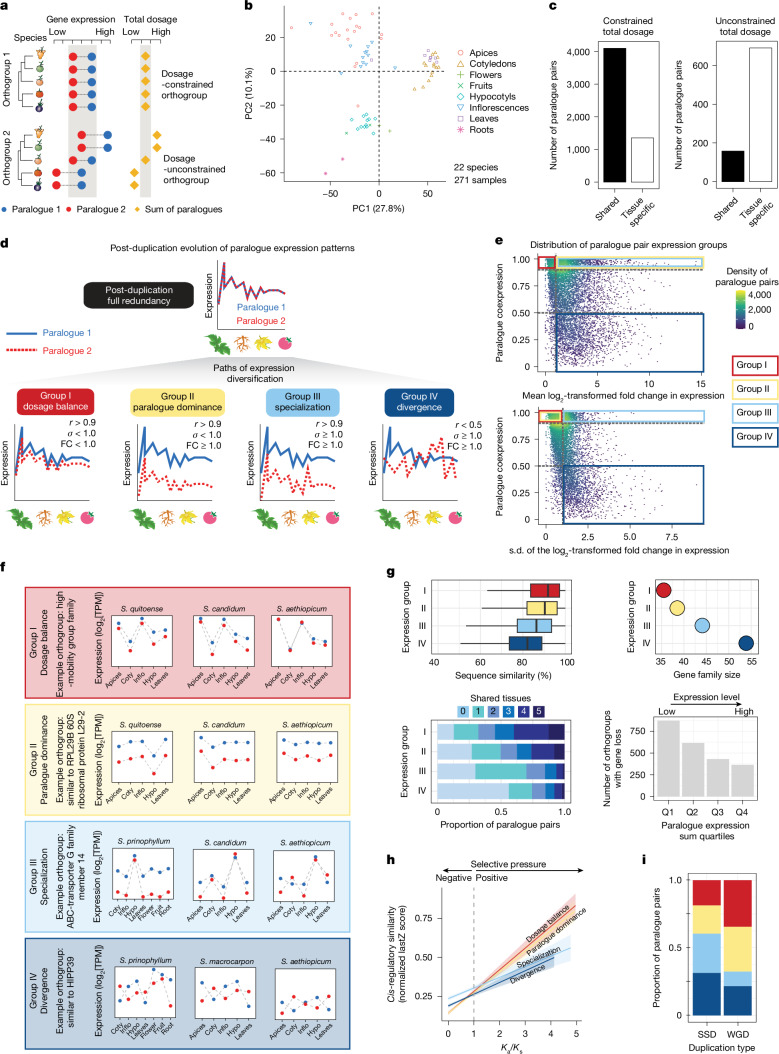
Widespread paralogous diversification across *Solanum* revealed by multitissue gene expression analysis. **a**, Schematic of dosage-constrained and dosage-unconstrained orthogroups reflecting different degrees of selection on the total dosage of paralogue pairs across species. **b**, PCA of the normalized expression matrix from 5,146 singleton genes shared across all 22 species. The expression matrix consists of the summed expression of paralogue pairs. Tissue samples are coloured by tissue identity. **c**, The tissue specificity of constrained and unconstrained paralogue pairs. Paralogue pairs under constrained total dosage across species are less tissue specific (left) than unconstrained paralogues (right). **d**, Schematic of four categories of functional expression groups of retained paralogues: group I, dosage balance; group II, paralogue dominance; group III, specialization; group IV, divergence. **e**, The distribution of paralogue pairs according to their co-expression level and mean log_2_[fold change (FC)] (top) or the s.d. of the log_2_[fold change] (bottom) in expression. The four derived paralogue expression groups are shown. **f**, Representatives of paralogue pairs capturing the different patterns of expression delimited across the pan-genome. Coty, cotyledon; hypo, hypocotyl; inflo, inflorescence. **g**, Genes included in the four paralogue expression groups display contrasting protein sequence similarity (top left), gene family size (top right), number of shared expression domains (tissues) (bottom left) or propensity to undergo gene loss for orthogroups in different dosage quartiles (bottom right). For all box plots, the box limits show the first and third quartiles, the centre line represents the median and the whiskers represent 1.5× the interquartile range. **h**, *Cis*-regulatory sequence conservation in the different expression groups in relation to increased selection on protein sequence. For each expression group, the predicted mean and 95% confidence interval of the normalized LastZ score is shown (details of the statistical analysis was provided in Supplementary Table 5). **i**, The proportion of each paralogue expression group attributed to paralogue pairs derived from either WGD or SSDs, showing increased divergence of paralogues from small-scale duplications.

**Extended Data Fig. 2:**
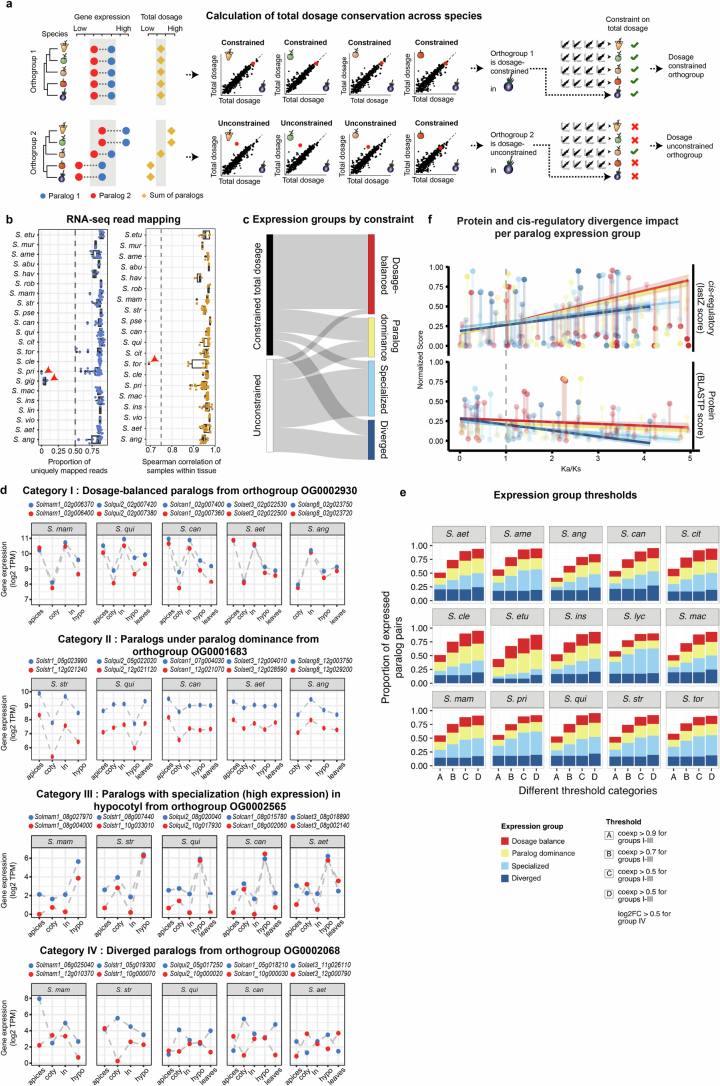
Paralog pairs expression analysis. **(a)** Schematic of dosage-constrained and dosage-unconstrained orthogroups reflecting different degrees of selection on the total dosage of paralog pairs across species. Orthogroup 1 has paralog pairs with identical total dosage across species, whereas orthogroup 2 has different total dosages in each species. For each tissue, orthogroup and species, the total dosage of two paralogs is compared with that of the two homologues in each of the remaining species, and deviations from the expected ratio of total dosages are classified as “unconstrained”. This is repeated for all species that share the orthogroup and expressed in the tissue of interest, and the majority classification across species is taken as the classification for the entire orthogroup. Therefore, orthogroup 1 is classified as “dosage-constrained” while orthogroup 2 is classified as “dosage-unconstrained”. **(b)** The fraction of uniquely mapped reads for each tissue sample and species (left), and the average gene expression correlation with other samples from the same tissue and species (right). Red arrows in both cases point to the five outlier samples excluded from further analysis. For all boxplots, the bounds of the box represent the first and third quartiles, the thick line represents the median and the whiskers represent 1.5× the interquartile range. **(c)** Sankey plot shows the concordance between classification of paralog pairs based on two independent approaches (total dosage conservation and conservation of expression levels and profiles). Thickness of lines connecting each pair of groups shows the odds ratio of enrichment. **(d)** Line plots showing examples of paralog pairs in each of the four groups of paralog expression patterns. **(e)** Proportion of expressed paralog pairs classified into one of four expression groups at different coexpression and fold-change thresholds in 15 species. Individual bars are coloured by expression groups. **(f)** Relationship of protein and *cis*-regulatory sequence conservation on the different paralog expression groups over increasing evolutionary pressure. For each expression group the predicted mean, 95% confidence interval, and residuals of the normalized LastZ score are shown (see Supplementary Table 5 for statistical analysis).

Research in yeast and other systems suggests that duplicated genes can negatively affect fitness due to increased expression dosage, which can lead to stoichiometric imbalances in macromolecular complexes^39,40^. Consequently, early diversification of *cis*-regulatory sequences may serve to restore ancestral single-copy gene dosage levels in a process called compensatory drift^28,41^. To explore constraints on total expression dosage from retained paralogues, we established two broad categories of paralogue pairs as dosage-constrained or dosage-unconstrained across species and on a per tissue basis (Fig. 2a). We defined dosage-constrained orthogroups as paralogue pairs that exhibited similar total expression levels in a given tissue across species, whereas dosage-unconstrained orthogroups did not maintain the same summed expression (Extended Data Fig. 2a). To assign paralogue pairs to these categories, we generated a pan-*Solanum* gene expression resource comprising 240 samples from 22 species, 15 of which had data from two or more distinct tissues (Extended Data Fig. 2b and Supplementary Table 6). Principal component analysis (PCA) of the transcripts-per-million (TPM)-normalized expression data of 5,146 singleton genes showed that the vast majority of samples clustered by tissue type (Fig. 2b). As in yeast^42^, our data show that paralogue pairs typically evolved under total dosage constraint across tissues and species (Fig. 2c). These pairs also exhibited much lower rates of non-synonymous mutations and were less likely to be tissue-specific than unconstrained pairs.

Dosage relationships between paralogue pairs can be influenced by different evolutionary trajectories resulting in divergent expression patterns. Among retained paralogue pairs within a given species, we considered four groups of common patterns of expression relationships after gene duplication (Fig. 2d and Extended Data Fig. 2c): group I, dosage balanced: selection on total dosage remains high, and pairs retain similar expression profiles and levels across tissues; group II, paralogue dominance: substantial divergence in expression levels that are proportional across tissues; group III, specialization: expression profiles no longer show a purely global shift and instead exhibit tissue-specific changes; group IV, divergence: paralogue pairs are fully diverged in both expression profile and level. Applying these definitions to our paralogue gene expression dataset assigned 58,130 paralogue pairs (around 53% of expressed paralogue pairs, 8% of total paralogue pairs) to a specific group (Fig. 2e,f and Extended Data Fig. 2d). A range of more relaxed parameters enabled up to 93% of expressed paralogues to be classified in these groups (Extended Data Fig. 2e).

While these groups were defined by the expression profiles across tissues within a species, the data also enabled us to evaluate whether the groups were associated with distinct genetic features. We compared protein sequence similarity between the groups, as well as gene family function, size, expression status, the number of tissues where expressed and transcription levels (Fig. 2g and Supplementary Fig. 5). Pairs in group I showed higher sequence similarity, smaller gene family size, broader expression across tissues and higher transcription levels compared with those in groups undergoing paralogue dominance, specialization and divergence (groups II–IV) (Fig. 2g). Functional enrichment analysis showed that groups I–II are enriched in dosage-sensitive processes such as transcription and translation, whereas groups III–IV are enriched, for example, in defence response genes (Extended Data Fig. 2e). Moreover, consistent with their conserved expression patterns, group I and II paralogue pairs maintained greater *cis*-regulatory sequence conservation than those in groups III and IV (Fig. 2h and Extended Data Fig. 2f).

We further reasoned that the type of duplications from which paralogue pairs originated might affect their expression relationships. We found that the most conserved expression groups (paralogue pairs in groups I and II that also capture more ancient duplications) were more likely to have originated from WGDs, whereas paralogue pairs in groups III and IV were enriched in small-scale duplications (SSDs) (Fig. 2i). Although paralogues in all four of our defined groups have the potential to complicate crop engineering, pairs with correlated expression patterns (groups I–III, 67% of classified paralogue pairs) pose the greatest challenge for translating knowledge between species owing to variable interdependent relationships that are redundant, compensatory or partially subfunctionalized. Overall, these analyses point to widespread paralogue emergence, expression change or loss in gene families spanning a multitude of biological functions, which has widespread implications for paralogues shaping genotype-to-phenotype relationships and species-specific contingencies in trait engineering.

## Genetics of paralogue diversification

**Fig. 3:**
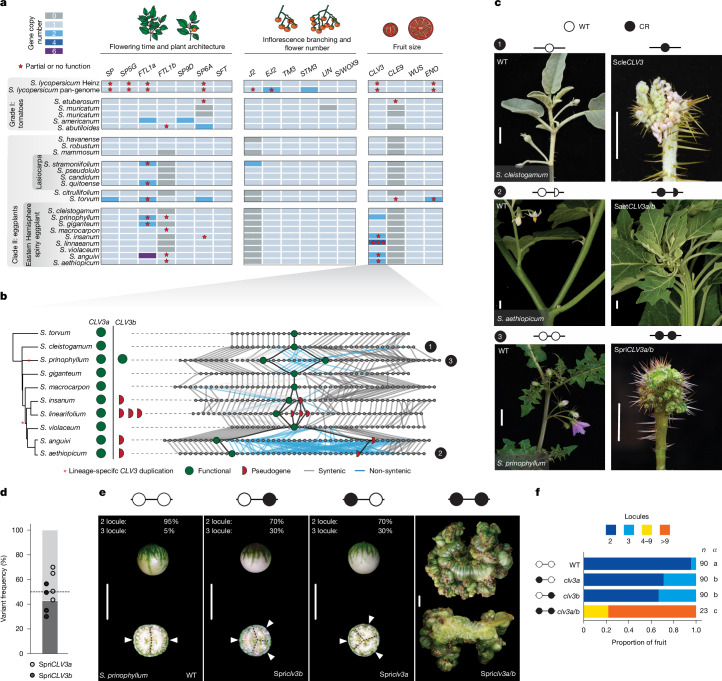
Functional dissection of lineage-specific paralogue diversification through pan-genetics reveals modified compensatory relationships in a major fruit size regulator. **a**, Pan-genome-wide gene presence/absence and copy-number variation in 17 orthogroups containing genes that are known to regulate three major domestication and improvement traits in tomato. The stars indicate partial or no gene function: hypomorphic allele or pseudogene. **b**, The haplotype diversification at the *CLV3* locus across the eggplant clade is substantial. The presence/absence of *CLV3* paralogues is shown. Lineage-specific *CLV3* duplications are marked with asterisks. The green full circles denote functional *CLV3* copies and the red half circles denote truncated/pseudogenized copies. The grey lines illustrate conservation, and the blue lines represent loss of synteny. **c**, CRISPR–Cas9 genome editing of *CLV3* orthologues in three species of the eggplant clade. Engineered loss-of-function mutations in *S. cleistogamum* (Scle*CLV3*, top), *S. aethiopicum* (Saet*CLV3a/b*, middle) and *S. prinophyllum* (Spri*CLV3a/b*, bottom) resulted in severely fasciated stems and flowers in all three species. Scale bars, 1 cm. **d**, Quantification of Spri*CLV3* paralogue-specific transcripts by RNA-seq. *n* = 4 biological replicates. **e**, Locules per fruit after paralogue-specific CRISPR gene editing of Spri*CLV3a* and Spri*CLV3b* in *S. prinophyllum*. Single paralogue mutants cause a subtle shift from bilocular to trilocular fruits; inactivation of both paralogues results in highly fasciated fruits. The arrowheads mark locules. Scale bars, 1 cm. **f**, Quantification of the locule number in single and double Spri*clv3a* and Spri*clv3b* mutants in *S. prinophyllum* showing paralogous *CLV3* dosage relationships. The proportion of each locule number per genotype is shown. *n* represents the number of fruits counted, *α* represents the statistically significant group. Source data and additional statistical information, including *P* values, are provided in Supplementary Tables 8 and 9.

The *Solanum* pan-genome provided an opportunity to study the extent to which paralogue diversifications have influenced genotype-to-phenotype relationships across the genus. On the basis of previous characterization and cloning of developmental genes and QTLs from model *Solanum* crops (primarily eggplant, potato and tomato), we compiled a set of 150 genes, and any associated paralogues, affecting 16 domestication and breeding traits (Supplementary Table 7). Our pan-genome revealed widespread variation in these genes both between and within clades, with numerous cases of presence–absence variation, copy-number variation, and gene truncation or pseudogenization across the pan-genome. All of these detected variations have the potential to affect predictability in engineering trait modifications, and prominent among these were 17 orthogroups that contribute to the three major components of crop domestication syndromes: (1) flowering time and plant architecture; (2) inflorescence architecture and flower number; and (3) fruit size (Fig. 3a and Supplementary Results).

Selection for increased fruit size in *Solanum* crops was a major driver of yield improvements. In tomato, this increase was largely facilitated by a promoter structural variant (SV) in the small signalling peptide gene *CLAVATA3* (*CLV3*), which represses stem cell proliferation in meristems^10^. This variant reduced *CLV3* expression and function, leading to an increase in stem cells, larger floral meristems and more floral organs, ultimately resulting in additional seed compartments (locules) in fruits. An ancestral, partially redundant paralogue of *CLV3*, known as *CLE9*, partially suppresses the increased locule number effect caused by the *CLV3* domestication allele^11,43^. In Solanaceae species in which both paralogues are retained^11^, *CLE9* falls into group II (paralogue dominance); however, in other species, *CLE9* was pseudogenized or completely lost, leaving *CLV3* without a partially redundant paralogue^11^.

**Extended Data Fig. 3:**
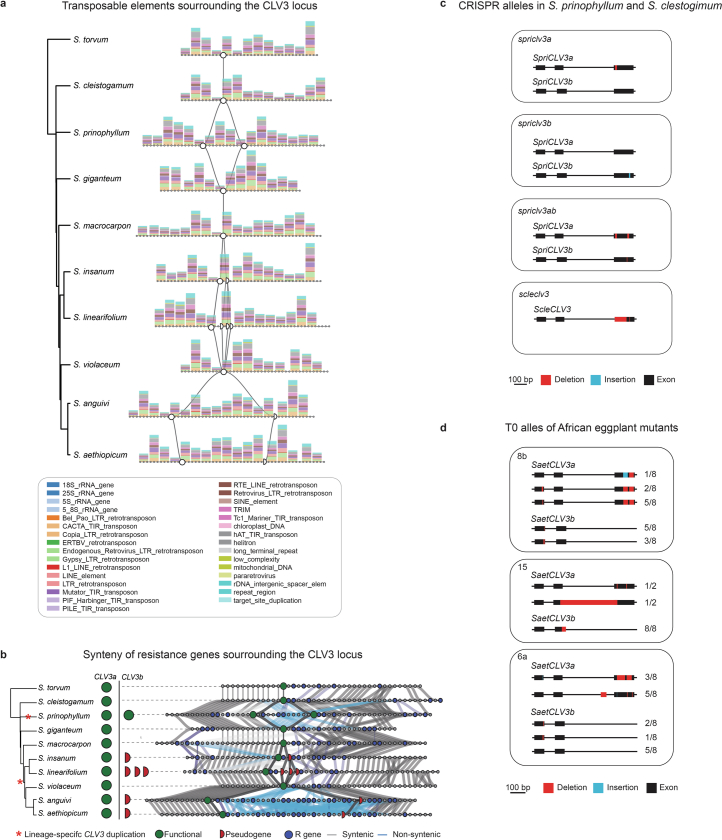
Extreme variation in transposable elements and resistant gene content at the *CLV3* locus across *Solanum.* **(a)** Gene and transposable element compositions are highly variable at the *CLV3* locus across the eggplant clade. While most of the gene content shows collinearity, the transposable element profile and density varies considerably. Stacked bars show the absolute number and type of transposable element for the window of three genes. **(b)** Microsyntenic relationships at the *CLV3* locus across the eggplant clade show dynamic expansions and contractions of resistance genes. Resistance genes are identified by blue dots. Presence-absence of *CLV3* paralogs is shown as in Fig. 3. Lineage-specific *CLV3* duplications denoted with asterisks. Window sizes range from 397,829 bp (*S. torvum*) to 634,079 bp (*S. aethiopicum*) and are centred on the *CLV3* locus. Functional *CLV3* copies are denoted by green full circles while truncated/pseudogenized copies are shown as red half circles, as in Fig. 3. Grey lines illustrate conservation, while blue lines represent loss of synteny. **(c)** CRISPR/Cas9 gene-edited loss-of-function null alleles of *CLV3* genes in *S. prinophyllum* and *S. cleistogamum*. **(d)** CRISPR/Cas9 gene-edited loss-of-function null alleles of African eggplant *SaetCLV3a/b*. Numbers represent the proportion of cloned and sequenced *SaetCLV3a/b* alleles as a ratio of the total number of clones sequenced in the three first-generation transgenic (T0) plants showing fasciation phenotypes.

In our *Solanum* pan-genome, we found that all species except for tomato and *Solanum americanum* either contain a pseudogenized *CLE9* or lack it entirely. Notably, despite this widespread loss of *CLE9*, a subset of the Eastern Hemisphere spiny eggplant clade possesses locally duplicated intact and pseudogenized copies of *CLV3* (Fig. 3a,b). Our chromosome-scale references revealed complex haplotypes involving these duplications, with species-specific transposable elements and disease-resistance genes interspersed between the paralogues. For example, whereas *Solanum prinophyllum* carries two intact copies of *CLV3*, one intact and a variable number of pseudogenized copies exist in *S. aethiopicum* (1 pseudogenized copy), its progenitor *Solanum anguivi* (1 pseudogenized copy) and *Solanum linnaeanum* (3 pseudogenized copies) (Fig. 3b and Extended Data Fig. 3a,b). Comparing these complex haplotypes and observing identical breakpoints in pseudogene structure across a subset of these species suggested at least two independent *CLV3* duplication events in the Eastern Hemisphere spiny clade. In the last common ancestor of *Solanum insanum*, *S. linnaeanum, S. anguivi* and *S. aethiopicum*, one duplication was followed by pseudogenization, whereas a more recent duplication emerged in the lineage leading to *S. prinophyllum* (Fig. 3b). However, as *Solanum violaceum* carries only one *CLV3* copy, we cannot exclude the possibility of three independent duplications.

The independent duplication that produced two intact copies of *CLV3* in *S. prinophyllum* suggests redundancy was re-established in this species, while in species in which one *CLV3* paralogue became pseudogenized, redundancy was again lost. We tested this by using CRISPR–Cas9 to inactivate *CLV3* in three spiny *Solanum* species: *Solanum cleistogamum* (desert raisin, Scle*CLV3* single copy), *S. aethiopicum* (African eggplant, one functional (Saet*CLV3a*) and one pseudogenized (Saet*CLV3b*)) and *S. prinophyllum* (intact copies of Spri*CLV3a* and Spri*CLV3b*) (Fig. 3c and Extended Data Fig. 3c,d). As expected, mutations in the one intact copy of *CLV3* in *S. cleistogamum* and *S. aethiopicum* led to extreme fasciation phenotypes, mirroring the severe phenotype in tomato *clv3* *cle9* double mutants (Fig. 3c). Similarly, knocking out both copies of *CLV3* in *S. prinophyllum* (Spri*CLV3a* and Spri*CLV3b*) resulted in the same severe fasciation.

Spri*CLV3a* and Spri*CLV3b* in *S. prinophyllum* are identical in their coding and *cis*-regulatory sequences, except for a single-nucleotide variant in the 3′ untranslated region of the ancestral copy. Such high sequence identity suggested that the elimination of one copy would be fully compensated by the remaining functional copy, similar to the near complete compensation between Pgri*CLV3* and Pgri*CLE9* in the Solanaceae species *Physalis grisea* (groundcherry)^11^. Our previously generated expression data from meristems of *S. prinophyllum*^44^ showed that both paralogues are expressed at similar levels (Fig. 3d), supporting this prediction. Notably, we found that engineered mutations in either of the Spri*CLV3* paralogues resulted in a higher percentage of trilocular fruits compared with the wild type (WT) (5% in the WT compared with 30% in single mutants), suggesting that one paralogue cannot fully compensate for the other, perhaps due to a gene expression dosage effect (Fig. 3e,f and Supplementary Table 8).

Taken together, these data suggest that, after the loss of the ancestral redundant *CLE9* paralogue, tandem duplication events in three spiny *Solanum* lineages probably reestablished *CLV3* compensation. However, this compensation was subsequently lost again in at least one lineage due to pseudogenization of the duplicated *CLV3* gene. Even in *S. prinophyllum*, in which two nearly identical copies of *CLV3* were retained, full compensation was either not achieved or not maintained.

## African eggplant paralogue diversification

**Fig. 4:**
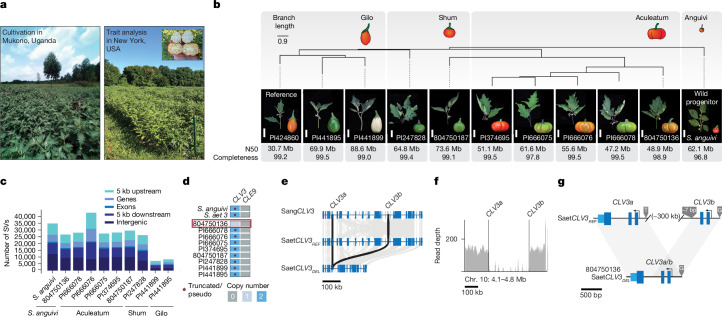
Pan-genome of African eggplant reveals widespread structural variation, wild species introgression and *CLV3* paralogue diversification. **a**, Images of field-grown African eggplant in Mukuno, Uganda (left) and New York, USA (right). **b**, Orthologue-based phylogeny of ten African eggplant accessions covering three main cultivar groups (Gilo, Shum and Aculeatum) and the wild progenitor *S. anguivi*. Representative shoots and fruits are shown for each accession. Scale bars, 5 cm (shoots). Genome summary statistics, including contig N50 (post-contamination screen) and post-assembly completeness^61^, are indicated. The branch lengths reflect coalescent units. **c**, The number of SVs overlapping with genomic features across accessions. **d**, The presence/absence of and copy-number variation in *CLV3* across the pan-genome. *CLE9* is absent in all genotypes. *S. aethiopicum* and *S. anguivi* are shown for reference. **e**, Conservation of exonic microsynteny (grey bars) between Sang*CLV3*, Saet*CLV3*_*REF*_ and Saet*CLV3*_*DEL*_ haplotypes. Scale bar, 100 kb. **f**, Long-read pile-up at the Saet*CLV3* locus identifies a deletion structural variation and a distinct Saet*CLV3* haplotype in accession 804750136. **g**, Diagram of a deletion–fusion allele of *CLV3* (Saet*CLV3*_*DEL*_) that arose in accession 804750136. The 7 bp indel and single-nucleotide polymorphisms (SNPs) were used as markers to validate the deletion–fusion scenario.

As exemplified by *CLV3*, dynamic duplication histories and the resulting species-specific variable functional relationships of paralogues (Fig. 3a) could have substantial effects on genome engineering outcomes when translating knowledge between crops, particularly when targeting gene families that are crucial in crop domestication and trait improvement. Within our pan-genome, African eggplant (*S. aethiopicum*) is a major crop indigenous to sub-Saharan Africa, cultivated across the continent on hundreds of thousands of acres. It is also important in Brazil, having been transported by enslaved Africans^45,46^ (Fig. 4a). Diverse cultivars are grown in Africa for their edible fruits or leaves, as well as for the ornamental appeal of specific fruit types^9^. The domestication history of African eggplant is largely unknown, but the species and its many cultivars exhibit broad intraspecific diversity in vegetative and fruit phenotypes, particularly fruit shape, colour and size, mirroring the wide diversity of tomatoes (Fig. 4b). Recent breeding efforts in African eggplant have primarily focused on adaptation to abiotic stress^47,48^, with less emphasis on improving productivity. Re-engineering or mimicking the effects of known beneficial mutations identified in tomato and other established *Solanum* model crops could rapidly improve yields. However, the limited availability of genomic and genetic resources leaves the extent to which background modifiers influence predictability in trait engineering unclear.

**Extended Data Fig. 4:**
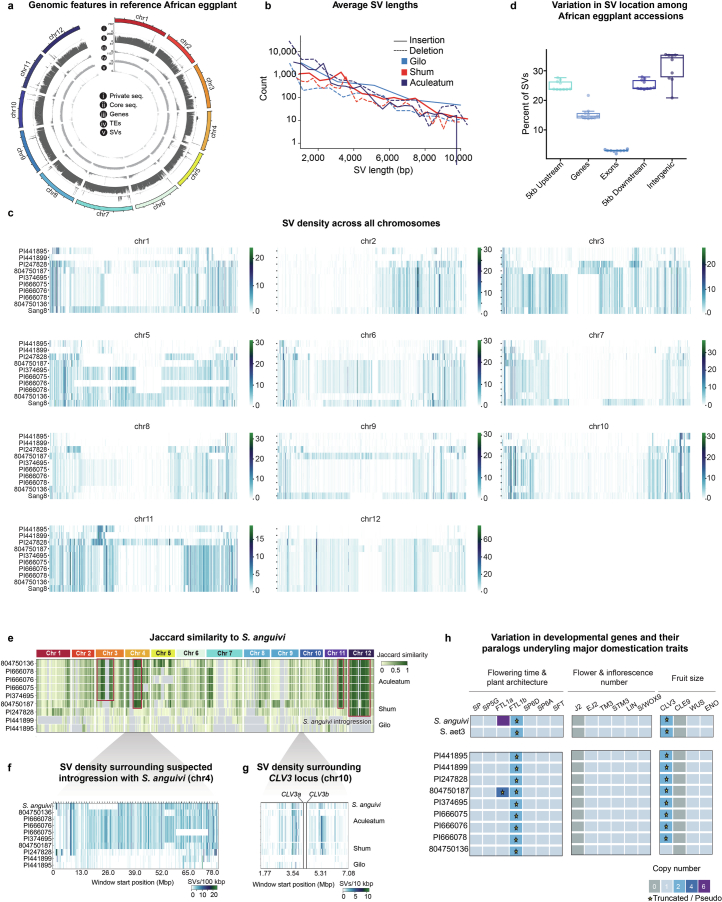
Structural variants and gene copy number variation in the African eggplant pan-genome. **(a)** Pan-genomic features across the African eggplant reference genome. Frequencies of: (i) sequences private to the reference, (ii) core sequence, (iii) genes, (iv) transposable elements, and (v) SVs. **(b)** Average SV lengths (bp) for deletions (dotted lines) and insertions (solid lines) across the three African eggplant cultivar groups. **(c)** Structural variant density across all chromosomes in African eggplant and its wild progenitor *S. anguivi* in 2 Mbp windows. **(d)** Percentage of structural variants overlapping with different genomic features. For all boxplots, the bounds of the box represent the first and third quartiles, the thick line represents the median and the whiskers represent 1.5× the interquartile range. **(e)** Jaccard similarity of SVs across the African eggplant pan-genome measured against *S. anguivi* in 2 Mbp windows. Putative introgression from *S. anguivi* on chromosomes 3, 4, 11, and 12 are highlighted by red boxes. **(f)** Close-up of chromosome 4 introgression shown by SV density. **(g)** SV density surrounding the *SaetCLV3* locus across the pan-genome. Genomic positions of *SaetCLV3a* and *SaetCLV3b* are shown. Window size: 10 kbp. **(h)** Gene presence-absence and copy number variation in 17 orthogroups containing known genes regulating three major domestication traits in tomato across the African eggplant pan-genome and *S. anguivi*. Stars mark gene truncation or pseudogenization.

To address this, we first phenotyped in field conditions eight representative accessions (Supplementary Table 10) from the Gilo (fruit production), Aculeatum (ornamental) and Shum (leaf production) cultivar groups (Fig. 4a), along with one accession of *S. anguivi*. On the basis of the observed phenotypic variation, we selected ten diverse accessions from the three groups and assembled a long-read-based African eggplant pan-genome that included its wild progenitor *S. anguivi* (Fig. 4b and Supplementary Tables 10 and 11). The African eggplant representative genotype in the *Solanum* pan-genome (Gilo accession PI 424860; Fig. 1) was selected as the reference genome. We computed an orthologue-based phylogenetic tree (Fig. 4b), which indicated two major clades, one comprising the three Gilo accessions and a second containing the five Aculeatum accessions. Notably, the two Shum accessions did not form a monophyletic group, suggesting that accessions cultivated for leaf production might have different genetic origins. Comparison of the African eggplant genomes with the reference revealed over 250,000 SVs, with variable densities genome-wide (Extended Data Fig. 4a–d and Supplementary Results). Similar to our tomato pan-genome^26^, over 68% of SVs were within 5 kb upstream or downstream of genes, in addition to 7,234 SVs overlapping exons and therefore likely to disrupt gene function (Fig. 4c, Extended Data Fig. 4c and Supplementary Results). These SVs also revealed several large introgressions from the *S. anguivi* wild ancestor, primarily in the Aculeatum group (Extended Data Fig. 4e,f and Supplementary Results).

As in tomato, African eggplant cultivar groups exhibit extreme variation in fruit size, based in part on variation in locule number (Fig. 4b). Recent diversification of key regulators of fruit locule number, such as Saet*CLV3*, might have favoured intraspecific phenotypic diversity. The Saet*CLV3* locus, located on chromosome 10, is nested in dense SV clusters (Extended Data Fig. 4g). Notably, we found one Aculeatum accession (804750136) with only a single intact copy of Saet*CLV3*, suggesting that the ancestral pseudogenized copy was lost (Fig. 4d and Extended Data Fig. 4h). Microsynteny analysis revealed broad rearrangements of the *CLV3* locus between African eggplant and *S. anguivi* as well as intraspecific diversity (Fig. 4e). We detected two deletions within the Saet*CLV3* locus in two *S. aethiopicum* accessions (804750136 and PI 247828), including a large approximately 300 kb deletion between the second exon of Saet*CLV3a* and the first exon of Saet*CLV3b* (Fig. 4f). Notably, this large deletion did not simply eliminate the Saet*CLV3b* pseudogene but, instead, resulted in a single fused functional copy of *CLV3*, which we designated Saet*CLV3*^*DEL*^ (Fig. 4g).

## Paralogues and African eggplant fruit size

**Fig. 5:**
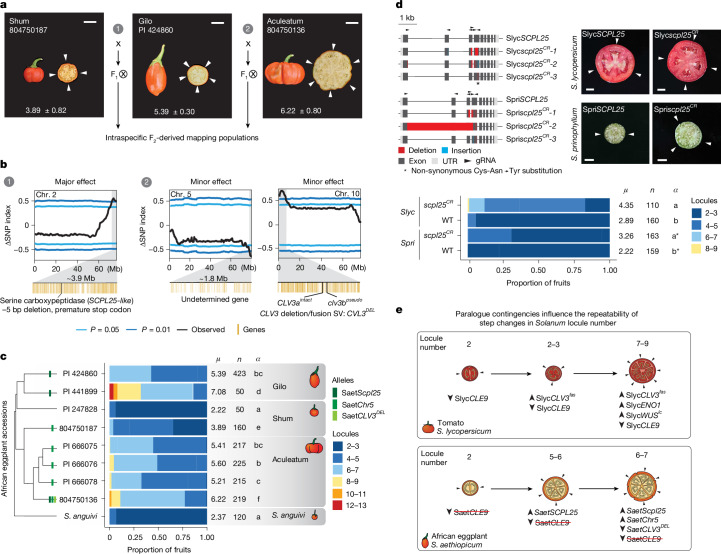
Pan-genetic dissection of fruit locule variation in African eggplant. **a**, Intraspecific crosses between representative accessions of each of the three main cultivated groups of African eggplant were used to generate F_2_ mapping populations for QTL-seq. Scale bars, 2 cm. **b**, Major-effect (1) and minor-effect (2) QTLs affecting the locule number, identified by bulk-segregant QTL-seq. ∆SNP indices for three identified QTL on chromosomes 2, 5 and 10 indicate the relative abundance of parental variants in bulked pools of F_2_ individuals (low- and high-locule classes) calculated in 2,000 kb sliding windows. **c**, The fruit locule number from phylogenetically arranged African eggplant accessions. The presence of the three mapped QTL alleles (different intensity green bars) in each accession is indicated on the phylogenetic tree. *n* represents the number of fruits counted, *μ* represents the average fruit locule number and *α* represents the statistically significant group. Source data and additional statistical information, including *P* values, are provided in Supplementary Tables 12 and 15. **d**, CRISPR–Cas9-engineered mutant alleles of *SCPL25* serine carboxypeptidase orthologues in tomato (Slyc*SCPL25*) and *S. prinophyllum* (Spri*SCPL25*) (left), along with representative images of transverse fruit sections from mutant plants (right) and quantification of fruit locule number (bottom), showing a consistent increase in fruit locule number across species. *n* represents the number of fruits counted, *μ* represents the average fruit locule number and *α* represents the statistically significant group. Source data and additional statistical information, including *P* values, are provided in Supplementary Tables 16 and 17. Scale bars, 1 cm. **e**, Schematics comparing the genetic basis of step changes underlying increased locule number and fruit size in tomato and African eggplant. The arrowheads in transverse fruit depictions indicate locules. The average fruit locule number (*μ*), fruit number (*n*) and statistically significant group (*α*) are indicated on the right of the stacked bar plots.

**Extended Data Fig. 5:**
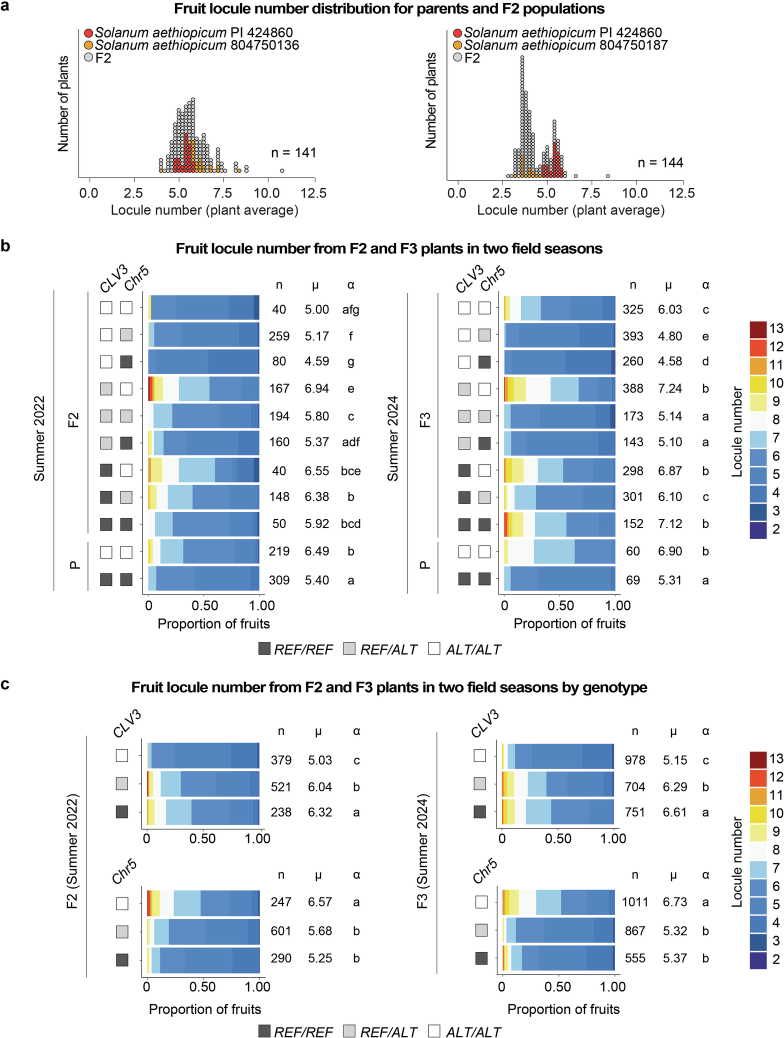
Interactions between the *CLV3* and Chr5 African eggplant locule number QTLs in F2 populations. **(a)** Mean fruit locule number for plants from the 804750136 × PI 424860 (left) and 804750187 × PI 424860 (right) derived segregating F2 populations grown in 2022 and used for QTL-seq analysis. Average locule counts for the parental genotypes are also shown. **(b)** Stacked bar plots showing fruit locule number from genotyped F2 (summer 2022, left) and F3 (summer 2024, right) plants derived from the 804750136 × PI 424860 cross. The genotyped reference (REF) and alternative (ALT) alleles of *SaetCLV3* and the chromosome 5 QTLs are presented. HET: heterozygous, P: parents. **(c)** Stacked bar plots as in (b) but showing the effects of alleles at each locus individually. Average fruit locule number (μ), fruit number (n) and statistically significant group (α) are indicated to the right of stacked bar plots. See Supplementary Tables 12–14, 18 and 19 for Source data and additional statistical information, including *p*-values.

We next evaluated whether these Saet*CLV3* paralogue evolutionary dynamics influenced locule number variation. Using our African eggplant genomes, we performed QTL-sequencing (QTL-seq) analysis to map loci controlling this trait (Supplementary Tables 12–14). We generated F_2_ mapping populations between the medium-locule count Gilo reference accession (PI 424860) and low- and high-locule count parents belonging to the Shum (804750187) and Aculeatum (804750136) groups, respectively (Fig. 5a and Extended Data Fig. 5a). In contrast to tomato, the major step change in locule number between the Gilo and Shum groups mapped to a QTL in a 3.9 Mb region on chromosome 2, which conspicuously did not include *CLV3* or any other known *CLV* pathway components (Fig. 5b). Instead, we identified a candidate gene encoding a serine carboxypeptidase (Saet*SCPL25-like* (*Solaet3_02g030160*), named after its best BLAST hit in *Arabidopsis*^49^) harbouring a 5 bp exonic frameshift deletion in the Gilo parent. Serine carboxypeptidases function in C-terminal peptide processing. Such control of CLE peptide processing has been demonstrated in *Arabidopsis*, in which mutation of the Zn^2+^ carboxypeptidase-encoding gene *SOL1* (*SUPPRESSOR OF LLP1*) represses CLE-dependent root meristem size-related defects^50^. The mutation in Saet*SCPL25-like* in the reference African eggplant accession was associated with approximately two additional fruit locules (Fig. 5c). Through CRISPR–Cas9 mutagenesis of the orthologues in both tomato (*Solyc02g088820*) and *S. prinophyllum* (*Solpri1_02g029870*), we validated this association and demonstrated a direct functional role of this gene in controlling locule number, resulting in increases in both species that are quantitatively similar to that of the natural mutation in African eggplant (Fig. 5d and Supplementary Table 16).

We also identified two minor-effect QTLs from the Aculeatum group that mapped to a 1.8 Mb region on chromosome 5 and a 4.9 Mb region on chromosome 10. Notably, the latter encompasses the Saet*CLV3*^*DEL*^ haplotype containing the reconstituted single functional copy of Saet*CLV3* (Figs. 4g and 5b). We found that Aculeatum parent alleles at the *CLV3* and chromosome 5 QTLs were associated with a decrease and increase in locule number, respectively (Extended Data Fig. 5b). These minor-effect QTLs were robust across years and environments, as confirmed by F_2_-derived F_3_ segregating populations (Extended Data Fig. 5b,c and Supplementary Table 18). While the specific gene(s) and variant(s) underlying the chromosome 5 QTL, along with its precise interaction with Saet*SCPL25-like* and *SaetCLV3*^*DEL*^, will require further characterization, our results indicate that at least three loci contribute to variation in locule number in African eggplants.

To better understand how these QTLs shaped the domestication history of African eggplant, we examined which alleles are present at the three identified loci within the phylogenetic context of our African eggplant pan-genome (Fig. 5c). The Gilo accessions contained the Saet*SCPL25-like* mutant allele, while the Aculeatum accessions and one of the Shum accessions contained the chromosome 5 minor-effect QTL’s haplotype. Meanwhile, a single Aculeatum accession (804750136) contained all three identified alleles, including the minor-effect Saet*CLV3*^*DEL*^ SV (Fig. 5c). The SV at Saet*CLV3* probably occurred secondarily to the mutation in Saet*SCPL25-like* and the chromosome 5 QTL. Saet*CLV3*^*DEL*^ reduces the locule number, and this epistatic interaction was perhaps selected to attenuate the increases in locule number conferred by the effects of Saet*SCPL25-like* and the chromosome 5 QTL (Extended Data Fig. 5b). This contrasts with tomato, in which a promoter SV impacting Sl*CLV3* (Slyc*CLV3*^*fas*^) is a widespread and major-effect QTL variant that more than doubles locule number, and is further enhanced and suppressed by other minor-effect QTLs, including the paralogue *SlCLE9*. Thus, while QTLs affecting *CLV* signalling are shared drivers of increased locule number in both tomato and African eggplant, the specific genes, alleles and epistatic interactions, as well as the magnitude and directionality of these individual and combined effects, are distinct (Fig. 5e). The recurrence of QTLs at *CLV3* in two independent domestication histories underscores the major contribution of structural variation in shaping paralogue evolutionary dynamics and parallel trajectories of crop domestication and improvement.

## Discussion

Plant pan-genome resources are emerging at an incredible pace^2^. These foundational resources should help to guide genome-editing approaches to advance translation of genotype-to-phenotype knowledge among related crops and their wild relatives^1,19^. However, decades of plant breeding have demonstrated that background genetic modifiers remain barriers to achieving predictable outcomes^21–23,51^. While sequencing high-quality plant references at scale, including potentially telomere-to-telomere genomes^52^, combined with forward genetics, can readily uncover background variation, identifying orthologues and paralogues and tracing their evolutionary trajectories remains an unsolved challenge. This challenge is compounded by the exceptionally complex history across flowering plants of ancient WDGs, subsequent lineage-specific fragmentation and more recent smaller-scale duplications.

Compared with pan-genomes of single species, pan-genomes spanning an entire genus or broader taxonomic scales can reveal more sequence variation and extreme cases of paralogue diversification. We followed an integrated process to address the challenge of resolving orthologues, paralogues and their diversification histories in the *Solanum* pan-genome. Our approach used existing annotations, augmented by multitissue RNA-seq de novo annotations and manual curation, to expose and compare ancient paralogues and recent tandem duplications. We mapped core and dispensable genes and, among the tens of thousands of paralogue pairs identified, expression analyses revealed a continuum of redundancy relationships, driven by drifting expression patterns, pseudogenization or gene loss. In particular, at least 67% of expressed paralogue pairs across nearly all biological functions fall into categories of expression diversification that have the potential to complicate targeted outcomes from breeding with natural or engineered mutations to improve agricultural traits. Notably, paralogues of the fruit-size gene *CLV3* spanned all three possible scenarios, caused by emergence and then loss of *CLE9*, independent tandem duplications of *CLV3*, extreme haplotype shuffling and *CLV3* pseudogenization, accounting for both within- and between-species variation in this major domestication trait. Our approaches and findings demonstrate how using knowledge from major crops to indigenous crops and wild species can reveal previously unknown factors driving trait variation and facilitate reciprocal knowledge exchange for crop improvement, including the identification of new genes for targeted trait modification. Furthermore, these integrated approaches reveal species- and genotype-specific functional relationships between genes and alleles, providing insights that enhance design strategies and improve predictability when breeding with natural or engineered variation.

Complex paralogue evolutionary histories undoubtedly affect the predictability of outcomes from genome engineering in nightshades, grasses, legumes and beyond. Assembling widely and deeply sampled species and genotypes into multilineage pan-genomes^37,53^ offers substantial opportunities to better understand the origins and frequencies of genome fragility within and between species, and to mobilize advances in machine learning for de novo genetic and genomic predictions at scale. As more accurate machine learning models are developed, micro-level analysis (for example, gene prediction, read-level basecalling^54^ or variant detection) as well as higher-level predictions of epigenomic and regulatory activity will continue to improve. Efforts to predict the effect of *cis*-regulatory variation on gene expression are also maturing, although limitations in the modelling frameworks and their training regimes remain obstacles to achieving high predictive accuracy^55^. Our study shows that such models must explicitly account for paralogues and their diversification dynamics over a wide range of evolutionary time scales. The ability to predict how genotype-to-phenotype relationships are influenced by paralogues and additional species-specific epistatic interactions will inevitably be enhanced through the development of foundation models trained on large catalogues of molecular, cellular and organismal data within and across species.

We also recognize that implementing pan-genomic and pan-genetic resources, tools and technologies requires a deeper understanding of—and sensitivity to—the central role that Indigenous knowledge and cultures have had in botany and agriculture^7,18,56^. Our work has greatly benefited from collaboration with local breeders, who guided the selection of lineages, species and cultivars of African eggplant. Continued knowledge sharing should expedite the effect of our pan-genome on agriculture, in particular the potential to accelerate yield improvements while simultaneously addressing the primary challenge of abiotic stress tolerance^57,58^. Our integrated genomic and genome-editing pipeline complements the rich genetic and phenotypic diversity available in the African eggplant germplasm, offering new and more predictable routes for breeding. For example, from dissecting the parallel, but distinct, genetic paths towards increased locule number in tomato and African eggplant, we have greater clarity in how to predictably increase locule number, fruit size and yield in this important crop.

We expect additional advances will come from resolving paralogue histories of flowering regulators, which have been central to the agricultural revolutions of many crops^6^. However, it is important to highlight that, while industrialized breeding emphasizes yield, the specific needs of subsistence farmers can be different^59^. For African eggplant, modifying the flowering time and inflorescence architecture are arguably as important as increasing fruit size. In varieties grown for fruit production, earlier flowering and more branched genotypes would dwarf plants while accelerating fruit production and total yield. Conversely, in varieties cultivated for leaf consumption, delayed flowering would extend vegetative growth and enhance vegetative yield^6,60^. We propose that the florigen–antiflorigen flowering hormone system, along with its MADS-box gene targets, should be the primary focus to achieve these breeding goals. Our analysis of African eggplant revealed distinct diversifications of both florigen and antiflorigen paralogues compared with patterns found in tomato^6^. Understanding these potential contingencies, in combination with pan-genome-enabled quantitative genetics, will facilitate predictable outcomes in genome engineering. Most paramount to the success of the next generation of breeding in indigenous crops is effective communication, productive collaboration and appreciation for the collective knowledge among local people, breeders, growers and scientists.

## Methods

### Plant material, phenotypic analyses and imaging

Details on all plant material used in this study, including the passport identification numbers of acquisitions from seed stock centres, are available in Supplementary Tables 1 and 10. All phenotypic assessments were performed on plants grown in greenhouses or fields. All of the images presented in all of the figures were taken by the authors and are our own. All illustrations (such as fruit representations) in all of the figures were prepared by the authors and are our own. Quantitative phenotypic data were collected manually in fields and greenhouses and recorded in Microsoft Excel. Source data are provided in Supplementary Tables 8, 12–14, 16 and 18. Seven herbarium vouchers were collected from field-grown *Solanum* accessions. Vouchers were deposited to the Steere Herbarium at the New York Botanical Garden (Supplementary Table 1).

### Tissue collection and high-molecular-mass DNA extraction

For extraction of high-molecular-mass DNA, young leaves were collected from 21-day-old light-grown seedlings. Before tissue collection, seedlings were etiolated in complete darkness for 48 h. Flash-frozen plant tissue was ground using a mortar and pestle and extracted in four volumes of ice-cold extraction buffer 1 (0.4 M sucrose, 10 mM Tris-HCl pH 8, 10 mM MgCl_2_ and 5 mM 2-mercaptoethanol). Extracts were briefly vortexed, incubated on ice for 15 min and filtered twice through a single layer of Miracloth (Millipore Sigma). Filtrates were centrifuged at 4,000 rpm for 20 min at 4 °C, and pellets were gently resuspended in 1 ml of extraction buffer 2 (0.25 M sucrose, 10 mM Tris-HCl pH 8, 10 mM MgCl_2_, 1% Triton X-100, and 5 mM 2-mercaptoethanol). Crude nuclear pellets were collected by centrifugation at 12,000*g* for 10 min at 4 °C and washed by resuspension in 1 ml of extraction buffer 2 followed by centrifugation at 12,000*g* for 10 min at 4 °C. Nuclear pellets were resuspended in 500 ml of extraction buffer 3 (1.7 M sucrose, 10 mM Tris-HCl pH 8, 0.15% Triton X-100, 2 mM MgCl_2_ and 5 mM 2-mercaptoethanol), layered over 500 ml extraction buffer 3 and centrifuged for 30 min at 16,000*g* at 4 °C. The nuclei were resuspended in 2.5 ml of nuclei lysis buffer (0.2 M Tris pH 7.5, 2 M NaCl, 50 mM EDTA and 55 mM CTAB) and 1 ml of 5% Sarkosyl solution and incubated at 60 °C for 30 min.

To extract DNA, nuclear extracts were gently mixed with 8.5 ml of chloroform:isoamyl alcohol solution (24:1) and slowly rotated for 15 min. After centrifugation at 4,000 rpm for 20 min, 3 ml of aqueous phase was transferred to new tubes and mixed with 300 ml of 3 M NaOAc and 6.6 ml of ice-cold ethanol. Precipitated DNA strands were transferred to new 1.5 ml tubes and washed twice with ice-cold 80% ethanol. Dried DNA strands were dissolved in 100 ml of elution buffer (10 mM Tris-HCl, pH 8.5) overnight at 4 °C. The quality, quantity and molecular mass of DNA samples were assessed using Nanodrop (Thermo Fisher Scientific), Qubit (Thermo Fisher Scientific) and pulsed-field gel electrophoresis (CHEF Mapper XA System, Bio-Rad) according to the manufacturer’s instructions.

### Genome assembly

Reference quality genome assemblies for each of the 22 species (and two reference quality genomes for *S. muricatum*) (accession information is provided in Supplementary Table 2) were generated using a combination of long-read sequencing (Pacific Biosciences) for contigging and optical mapping (Bionano Genomics) for scaffolding. Between 1 and 4 PacBio Sequel IIe flow cells (Pacific Biosciences) were used for the sequencing of each sample in the *Solanum* wide pan-genome (average read N50 = 29,067 bp, average coverage = 63×). The exact number of flow cells and sequencing technology for each sample are provided in Supplementary Table 2. For the additional nine *S. aethiopicum* samples, a combination of PacBio Sequel IIe, PacBio Revio sequencing and Oxford Nanopore sequencing was used to assemble the genomes (Supplementary Table 11). Before assembly, we counted *k*-mers from raw reads using KMC3^62^ (v.3.2.1) and estimated the genome size, sequencing coverage and heterozygosity using GenomeScope (v.2.0)^63^. For five samples (details are provided in Supplementary Table 2), low-quality reads were filtered out with a custom script (https://github.com/pan-sol/pan-sol-pipelines). Sequencing reads from each sample were assembled using hifiasm^64^ and the exact parameters and software version varied between the samples based on the level of estimated heterozygosity and are reported in Supplementary Table 2. After assembly, the draft contigs were screened for possible microbial contamination as previously described^26^. Nchart was generated with ggplot2 (https://ggplot2.tidyverse.org/) using adaptation of N-chart (https://github.com/MariaNattestad/Nchart).

### Genome assembly scaffolding

Optical mapping (Bionano Genomics) was performed for 17 samples to facilitate scaffolding. Scaffolding with optical maps was performed using the Bionano solve Hybrid Scaffold pipeline with the recommended default parameters (https://bionano.com/software-downloads/). Hybrid scaffold N50s ranged from 33,254,022 bp to 219,385,699 bp (further details, including Bionano molecules per sample, are provided in Supplementary Table 2). High-throughput chromosome conformation capture (Hi-C) from Arima Genomics was performed for eight samples to finalize scaffolding. With Hi-C, reads were integrated with the Juicer (v.0.7.17-r1198-dirty) pipeline. Next, misjoins and chromosomal boundaries were manually curated in the Juicebox (v.1.11.08) application. Chromosomes were named based on sequence homology, determined using the RagTag^65^ scaffold (v.2.1.0, default parameters), with the phylogenetically closest finished genome (Supplementary Table 2), 12 of these samples (including nine *S. aethiopicum* samples) were scaffolded with Ragtag. Finally, small contigs (<50,000 bp) with >95% of the sequence mapping to a named chromosome were removed. Moreover, small contigs (<100,000 bp) with >80% of the sequence mapping to a named chromosome that contained one or more duplicated BUSCO genes, but no single BUSCO genes, were also removed using a Python script. Using merqury^61^ with the HiFi data, the final consensus quality of the assemblies was estimated as QV = 53 on average and a completeness of 99.2741% on average.

### Tissue collection, RNA extraction and quantification

All tissues were collected in 3–4 biological replicates from different greenhouse-grown plants at approximately 09:00–10:00 and flash-frozen in liquid nitrogen in 1.5 ml microfuge tubes containing a 5/32 inch (about 3.97 mm) 440 stainless steel ball bearing (BC Precision). Tubes containing tissue were placed in a −80 °C stainless steel tube rack and ground using a SPEX SamplePrep 2010 Geno/Grinder (Cole-Parmer) for 1 min at 1,440 rpm. For shoot apices, total RNA was extracted using TRIzol (Invitrogen) according to the manufacturer’s instructions for ground tissue. For all other tissues (cotyledons, hypocotyls, leaves, flower buds and flowers), total RNA was extracted using Quick-RNA MicroPrep Kit (Zymo Research). RNA was treated with DNase I (Zymo Research) according to the manufacturer’s instructions. The purity and concentration of the resulting total RNA was assessed using the NanoDrop One spectrophotometer (Thermo Fisher Scientific). Libraries for RNA-seq were prepared using the KAPA mRNA HyperPrep Kit (Roche). Paired-end 100 base sequencing was conducted on the NextSeq 2000 P3 sequencing platform (Illumina). Reads were trimmed using trimmomatic (v.0.39)^66^ and then mapped to their respective genome using STAR (v.2.7.5c)^67^ and expression was computed in TPM.

### Gene annotation

The gene-annotation pipeline (Supplementary Fig. 2c) involved several crucial steps, beginning with lift over of gene models using the Liftoff algorithm on community-established references of tomato (Heinz reference genome) and eggplant (Brinjal reference genome). We augmented the annotation using RNA-seq data from 15 species and multiple tissues for de novo annotation. Initially, the quality of raw RNA-seq reads from each sample (Supplementary Table 6) underwent assessment using FastQC v.0.11.9 (http://www.bioinformatics.babraham.ac.uk/projects/fastqc/). Subsequently, reference-based transcripts were generated using the STAR (v.2.7.5c)^67^ and Stringtie2 (v.2.1.2)^68^ workflows. To refine the data, invalid splice junctions from the STAR aligner were filtered out using Portcullis (v.1.2.0)^69^. Orthologues with coverage above 50% and 75% identity were lifted from the tomato reference genome Heinz (v.4.0)^70^ and the eggplant reference genome Eggplant (v.4.1)^71^ using Liftoff (v.1.6.3)^72^ using the parameters --copies,--exclude_partial and using both the Gmap (v.2020-10-14)^73^ and Minimap2 (v.2.17-r941)^74^ aligners. Furthermore, protein evidence from several published Solanaceae genomes^70,71,75^, and the UniProt/SwissProt database were used to support gene annotation. Structural gene annotations were generated using the Mikado (v.2.0rc2)^76^ framework, leveraging evidence from the Daijin pipeline. Moreover, microsynteny and shared orthology to Heinz v.4.0 and Eggplant v.4.0 were assessed using Microsynteny and Orthofinder (v.2.5.2)^77^. Correction of gene models with inframe stop codons was performed using Miniprot2^78^ protein alignments to incorporate protein data from Heinz v.4.0 and Eggplant v.4.1. Furthermore, gene models lacking start or stop codons were adjusted by placing them within 300 bp of the nearest codon location using a custom Python script (https://github.com/pan-sol/pan-sol-pipelines). Overall gene synteny was visualized using GENESPACE (v.1.3.1)^79^.

For functional annotation, ENTAP (v.0.10.8)^80^ integrated data from diverse databases such as PLAZA dicots (v.5.0)^81^, UniProt/Swissprot^82^, TREMBL, RefSeq, Solanaceae proteins and InterProScan5^83^ with Pfam, TIGRFAM, Gene Ontology and TRAPID^84^ annotations. Finally, the annotated data underwent a series of filtering steps, excluding proteins shorter than 20 amino acids, those exceeding 20 times the length of functional orthologues and transposable element genes, which were removed using the TEsorter^85^ pipeline.

We assessed the completeness of the gene models by assessing single-copy orthologues through BUSCO^86^ in protein mode, comparing them against the solanales_odb10 database (Supplementary Tables 2 and 3). Moreover, we examined the presence or absence of a curated set of 150 candidate genes known to be relevant in plant development and QTL studies (Supplementary Table 7).

### Transposable element annotation

The *S. lycopersicum* chloroplast and mitochondrion sequences were collected from NCBI reference sequences NC_007898.3 and NC_035963.1, respectively. Non-transposable-element repeat sequences, including 18S rDNA (OK073663.1), 5S rDNA (X55697.1), 5.8S rDNA (X52265.1), 25S rDNA (OK073662.1), DNA spacer (AY366528.1), centromeric repeat (JA176199.1) and telomere sequences (TTTAGGG), were collected from the NCBI and further curated. Transposable element sequences curated in the SUN locus study^87^ as well as several other transposable element sequences from NCBI were also collected. These sequences were combined as the curated set of tomato repeats.

De novo transposable element annotation was first performed on each genome using EDTA (v.2.1.5)^88^, with coding sequences from the ITAG4.0 Eggplant V4 annotation^89^ provided (--cds) to purge gene coding sequences in the transposable element annotation and parameters of --anno 1 --sensitive 1 for sensitive detection and annotation of repeat sequences. Curated tomato repeats were supplied to EDTA (--curatedlib) for de novo annotation. Transposable element annotations of individual genomes were together processed by panEDTA^90^ for the creation of consistent pan-genome transposable element annotation. The summary of whole-genome repeat annotations was derived from .sum files generated by panEDTA (Supplementary Table 4).

Evaluation of repeat assembly quality was performed using LAI (b3.2)^91^ with inputs generated by EDTA and parameters -t 48 -unlock. LAI of *S. aethiopicum* genomes were standardized based on the HiFi-based reference assembly, with the parameters -iden 95.71 -totLTR 49.22 -genome_size 1102623763 -t 48 -unlock.

### Generation of CRISPR–Cas9-induced mutants

CRISPR guide RNAs to target *CLV3* and *SCPL25* across *Solanum* species were designed using Geneious (listed in Supplementary Table 20). The Golden Gate cloning approach was used to create multiplexed gRNA constructs. Plant regeneration and *Agrobacterium tumefaciens*-mediated transformation of *S*. *prinophyllum* were performed according to our previously published protocol^92^. For *S. cleistogamum* plant regeneration, the medium was supplemented with 0.5 mg l^−1^ zeatin instead of 2 mg l^−1^ and, for the selection medium, 75 mg l^−1^ kanamycin was used instead of 200 mg l^−1^. For *S. aethiopicum*, the protocol was the same as for *S. cleistogamum*, except the fourth transfer of transformed plantlets was done onto medium supplemented with 50 mg l^−1^ kanamycin. The seed germination time in culture can vary between species and batches of harvested seeds. Typically, *S*. *prinophyllum* germination took 8–10 days, *S*. *cleistogamum* germinated in 6–8 days and *S. aethiopicum* in 7–10 days.

### Distribution maps and species status

Species were categorized into wild, domesticated, locally important consumed or ornamental based on taxonomic literature and expert opinion^17^ (PBI *Solanum* Project (2024), Solanaceae Source; http://www.solanaceaesource.org/). The distribution maps were generated using the open source osm-liberty package (http://github.com/maputnik/osm-liberty/). Native ranges were derived from the same taxonomic literature and approximate centroids of the ranges were used for the mapping. The map is from osm-liberty, designed for open source maps.

### Phylogenomic analyses

*Jaltomata sinuosa*^93^ was used as an outgroup for the *Solanum* pan-genome tree, whereas the closely related *S. anguivi*, *S. insanum* and *S. melongena* were used as an outgroup for the *S. aethiopicum* dataset. Orthofinder^77^ was used to identify single-copy orthologues across all species. This resulted in 7,825 loci for the *Solanum* pan-genome dataset, and 19,769 loci for the *S. aethiopicum* dataset. To reduce the computing time, we randomly subsampled 5,000 loci for the *S. aethiopicum* dataset. This strategy was validated by topology, bootstrap support and gene tree concordance factors that are nearly identical to results obtained from a smaller 353 loci dataset described previously^35^. To reduce the effect of missing data and long branch attraction, sequences shorter than 25% of the average length for each loci were eliminated as described previously^35^. MAFFT^94^ was used to align each locus individually. Only loci that had all species in the alignment were retained. trimAl was also used to remove columns that had more than 75% gaps. IQ‐TREE2 (ref. ^95^) was used to generate individual ML trees for each locus. The resulting phylogenies were used for coalescent analyses with ASTRAL‐III (v.5.7.3)^96^, where tree nodes with <30% BS values were collapsed using Newick Utilities (v.1.5.0)^97^. Branch support was assessed using localPP support^98^, where PP values > 0.95 were considered strong, 0.75 to 0.94 weak to moderate, and ≤0.74 as unsupported. Trees were visualized with R using the packages ggtree^99^ and treeio^100^.

The 22 *Solanum* species were distributed into two major clades, grade I and clade II, along an orthologue-based phylogenetic tree. The terms grade I and clade II are established clade names in *Solanum*, originating from reference phylogenetic publications^35^. These were formally referred to as clade I and clade II, but clade I was shown to consist of a set of paraphyletic clades that do not form a monophyletic group. Thus, they are now referred to as grade I to reflect their evolutionary origin.

### Gene expansion contraction analysis

To analyse gene expansions and contractions, we processed the ultrametric species tree and gene family counts from OrthoFinder using CAFE5 (ref. ^101^). CAFE5 was run with the gamma model and parameter ‘k = 3’ to identify changes in gene family size along the species tree while accounting for rate variation among gene families.

### GO enrichment analysis

Gene Ontology (GO) enrichment analysis was performed using the GOATOOLS package^102^ to investigate the functional implications of genes associated with various duplication types including whole-genome (WGD), tandem (TD), proximal (PD), transposed (TSD) and dispersed (DSD) duplications. Genes were classified into these different duplication categories by DupGen_finder^38^. Moreover, we conducted GO enrichment on gene expansions (Supplementary Table 21) and contractions (Supplementary Table 22) identified across all lineages as reported by CAFE5, to examine functional trends related to these gene copy-number changes across the pangenome.

### Synteny analysis

The genomic neighbourhood around *CLV3* for selected species was manually inspected to detect and annotate intact and pseudogenized *CLV3* copies using pairwise sequence comparison with Exonerate (www.ebi.ac.uk/about/vertebrate-genomics/software/exonerate). Synteny plots were generated from a reciprocal BLASTP table obtained running Clinker (v.0.0.29, github.com/gamcil/clinker). Pseudomolecule visualization was generated via a custom script (https://github.com/pan-sol/pan-sol-pipelines). Transposable elements and resistance genes annotations were overlaid as needed using custom scripts (https://github.com/pan-sol/pan-sol-pipelines).

### Gene expression analysis

Reads from each tissue sample were aligned to the corresponding species-specific genome using STAR (v.2.7.2b)^67^, and only samples with more than 50% uniquely mapped reads were retained for subsequent analysis. For each species with two or more biological replicates per tissue, we calculated the Spearman correlation between tissue replicates, and removed samples with low correlation (0.75 or below). This yielded gene expression estimates for 240 samples across 22 species, with 15 species having expression data in two or more tissues. Specifically, 7 out of 22 species had expression data exclusively from the apex tissue, while 15 species had expression from two or more tissues. As expression diversification groups are defined based on the coexpression and expression fold change of paralogue pairs across two or more tissues, the analyses focused on 15 out of 22 species. Expression data were TPM-normalized and genes with zero expression across all of the samples were excluded from further analysis. PCA was performed on the tissue-specific expression profiles of 5,146 singleton genes selected based on Orthofinder results and shared across all 22 species to reveal the global relationships among samples. Plotting was performed using ggplot2 (https://ggplot2.tidyverse.org/). This validated the expected results that expression was largely clustering by tissue type.

### Analysis of whether the total dosage of duplicate gene pairs is conserved across *Solanum*

Survival of a gene after duplication depends on the competition between preservation to maintain partial or total dosage and mutational degradation rendering one copy with reduced or no function. Consequently, functional fates of duplicate genes are often characterized by the extent of selective pressures on total dosage. To assess the relative importance of dosage balance (copies evolving under strong purifying selection to maintain total dosage) and neutral drift (no selection on total dosage) in maintaining duplicate genes, we compared the total expression of paralogue pairs within each tissue for each pair of species. Note that the prickle tissue from *S. prinophyllum* is not included in this analysis as it is absent in the other 21 species.

In each tissue, gene expression was averaged over the biological replicates for each species. For each pair of species with expression data in a shared tissue, orthogroups with exactly two copies in each species with non-zero average expression in the tissue were retained for further analysis. For each tissue and species pair, we calculated the summed expression of paralogue pairs in each retained orthogroup, and observed that the total orthogroup-level expression was highly correlated across species, suggesting a prominent role of dosage balance in shaping the expression evolution of paralogues. We computed the ratio of the orthogroup-level expression between the species pair and transformed them into *z* scores. For each orthogroup in a species expressed in the tissue of interest, we averaged the *P* values from all pairwise species comparisons, adjusted the average *P* values using Benjamini–Hochberg correction and classified orthogroups with an adjusted average *P* < 0.05 as dosage-unconstrained orthogroups. All other orthogroups in the species and tissue were assumed to be evolving under constraint on total dosage.

All other orthogroups were assumed to evolve under selective constraint on total dosage. Note that the high *z*-score threshold provides a conservative estimate of the number of paralogue pairs evolving under drift. Sequence evolution rates for paralogue pairs (*K*_a_/*K*_s_) were calculated using KaKs_Calculator (v.2.0)^103^.

### Different modes of paralogue functional evolution

For each of the 15 species in which expression data were collected for two or more tissues, the expression data were first subset to genes with greater-than-median expression in at least one sample. The coexpression network for each species was constructed by calculating the Pearson correlation between all pairs of genes, ranking the correlation coefficients for each gene (with NAs assigned the median rank) and then standardizing the network by the maximum ranked correlation coefficient. From OrthoFinder, we obtained 763,492 paralogue pairs across the 15 species, representing all combinations of gene pairs within orthogroups. Of these pairs, 71% had low or no expression, and another 15% were filtered out due to insufficient expression for reliable analysis. This left 14% of pairs for further classification, where 8% (57% out of the 14% available for further classification) fit into one of four expression diversification groups below, while the remaining 6% did not meet our thresholds. Coexpression for each pair of paralogues in each orthogroup was obtained from this rank-standardized network. For each paralogue pair with non-zero expression in two or more samples, we also computed the fold change in expression across samples and used the absolute values of mean and s.d. of log_2_-transformed fold change across samples to summarize the degree of expression divergence between the two copies.

We classified the paralogue pairs within each species into different retention categories based on their variation in expression levels and correlated expression across samples. We selected these two axes of variation as they intuitively represent average expression difference (fold change) and specific pattern of difference (coexpression) between gene pairs. We classified paralogue pairs into four broad groups as follows:

(I) Dosage balanced: coexpression > 0.9; mean log_2_[fold change] < 1, s.d. of log_2_[fold change] < 1.
(II) Paralogue dominance: coexpression > 0.9; mean log_2_[fold change] ≥ 1, s.d. of log_2_[fold change] < 1.
(III) Specialized: coexpression > 0.9; mean log_2_[fold change] ≥ 1; s.d. of log_2_[fold change] ≥ 1.
(IV) Diverged: coexpression < 0.5, mean log_2_[fold change] ≥ 1; s.d. of log_2_[fold change] ≥ 1.

Paralogues originating from whole-genome, tandem and proximal duplications were obtained using the DupGen_finder pipeline^38^. WGD pairs with *K*_s_ ranging from 0.2 to 2.5, and tandem and proximal duplicates with *K*_s_ ranging from 0.05 to 2.5 were used to generate the stacked bar plots corresponding to WGDs and SSDs, respectively, in Fig. 2i.

The gene family size for each classified paralogue pair within a species corresponds to the number of genes in its orthogroup. The expression breadth of a gene corresponds to the number of tissues (among apices, cotyledon, hypocotyl, inflorescence, leaves) where the gene has an average expression greater than 3 TPM. The number of shared tissues expressing a paralogue pair is computed by intersecting the expression breadths of both copies, and ranges from 0 to 5. A gene was considered non-functional if it was annotated as a pseudogene or had an average expression below 3 TPM. Tissue-specific genes for each tissue were identified as genes with the highest expression in the tissue of interest, tissue-specificity score^104^ greater than 0.7 and with expression greater than 5 TPM in the relevant tissue. Both tissue specificity and pseudogene calling are sensitive to the breadth of tissue sampling, and the collection and incorporation of additional data into this framework would improve the comprehensiveness of the calling of modes of paralogue evolution.

### Mapping of loci controlling the *S. aethiopicum* locule number

The high-locule-count parent and reference accession PI 424860, and low- and higher-locule-count parents 804750187 and 804750136, respectively, were selected as founding parents to map QTLs and their causative variants affecting fruit locule number. Resulting F_1_ progeny were selfed to generate F_2_ mapping populations, which were sown in the greenhouse and then transplanted to a field site at Lloyd Harbor, New York, USA, during the summer of 2022. Six F_3_ populations derived from genotyped (see below) F_2_ individuals were sown and transplanted at the same location during the summer of 2024. Approximately ten fruits were collected from each F_2_ individual and the number of locules exposed by slicing each fruit transversely and counting. In the F_2_ populations derived from 804750187 × PI 424860 and 804750136 × PI 424860, 144 and 135 individuals were phenotyped, respectively (Supplementary Tables 13 and 14). For each population, DNA from 30 random individuals at the low and high ends of the phenotypic distribution for locule number were pooled for bulk-segregant QTL-seq analysis. The DNA from eight individuals of the common parental accession PI 424860 were also pooled to capture parental polymorphisms.

DNA from 15 of the most extreme low- and high-locule count individuals was extracted from young leaf tissue using the DNeasy Plant Pro Kit (Qiagen) according to the manufacturer’s instructions for high-polysaccharide-content plant tissue. Tissue used for extraction was ground using a SPEX SamplePrep 2010 Geno/Grinder (Cole-Parmer) for 2 min at 1,440 rpm. The sample DNA (1 µl assay volume) concentrations were assayed using Qubit 1× dsDNA HS buffer (Thermo Fisher Scientific) on the Qubit 4 fluorometer (Thermo Fisher Scientific) according to the manufacturer’s instructions. Separate pools were made for the parents, the bulked high-locule-count F_2_ individuals and the bulked low-locule-count F_2_ individuals, with an equivalent mass of DNA pooled from each individual to yield a final pooled mass of 3 µg in each bulk. DNA pools were purified using 1.8× volume of AMPure XP beads (Beckman Coulter) and the DNA concentration and purity were assayed using Qubit and the NanoDrop One spectrophotometer (Thermo Fisher Scientific), respectively.

Paired-end sequencing libraries for QTL-seq analysis were prepared with >1 µg of DNA using the KAPA HyperPrep PCR-free kit (Roche) according to the manufacturer’s instructions. Indexed libraries were pooled for sequencing on a NextSeq 2000 P3 chip (Illumina). Mapping was performed using the end-to-end pipeline implemented in the QTL-seq software package^105^ (v.2.2.4, https://github.com/YuSugihara/QTL-seq) with reads aligned against the *S. aethiopicum* (Saet3, PI 424860) genome assembly.

To determine the effects of the two identified QTL on locule number in the populations derived from 804750136 × PI 424860, co-segregation analysis was performed on the full F_2_ populations by genotyping Saet*CLV3* and the minor-effect locus on chromosome 5. For Saet*CLV3*, a cleaved amplified polymorphic sequence (CAPS) assay was used to genotype a variant in the promoter region of Saet*CLV3* linked to the identified *CLV3* SV haplotypes. A 1,258 bp region surrounding an AseI restriction fragment length polymorphism in the Saet*CLV3* promoter was amplified using the KOD One PCR Master Mix (Toyobo) on template DNA extracted using the cetyltrimethylammonium bromide method^106^ (primers 5431 and 4681 are shown in Supplementary Table 20). To 5 µl of the resulting PCR product, a 10 µl reaction containing 0.2 µl AseI (New England Biolabs) and 1 µl CutSmart r3.1 buffer (New England BioLabs) was incubated for 2 h at 37 °C. The reactions were then loaded onto a 1% agarose gel and electrophoresed in an Owl D3-14 electrophoresis box (Thermo Fisher Scientific) containing 1× TBE buffer for 30 min at 180 V delivered from an Owl EC 300 XL power supply (Thermo Fisher Scientific). The electrophoresis results were visualized under UV light using the Bio-Rad ChemiDoc XRS+ (Bio-Rad) imaging platform and ImageLab (Bio-Rad) software. The resulting banding patterns were then used to assign genotypes. For the chromosome 5 QTL, primers (primers 5883 and 5884 are shown in Supplementary Table 20) were used to amplify a 425 bp region containing a 1 bp deletion occurring near the summit of the QTL peak using the KOD One PCR Master Mix. The resulting PCR products were purified using Ampure 1.8× beads and were used as a template for Sanger sequencing (Azenta Genewiz). The sequencing results were then used to assign genotype calls at chromosome 5. Presented data are from individuals that were successfully genotyped at both loci.

### Conservatory analysis

The Conservatory algorithm (v.2.0)^107^ was used to identify conserved non-coding sequences (CNSs) within the Solanaceae family (Supplementary Fig. 4b) (https://conservatorycns.com/dist/pages/conservatory/about.php). A total of 26 genomes, including 23 *Solanum* genomes, two tomato genomes (Heinz and M82) and one groundcherry (*P. grisea*), were used as references to enable the identification of CNSs irrespective of structural variations among references. Protein similarity was scored using Bitscore^108^, while *cis*-regulatory similarity was assessed using LastZ^109^ score. Homologous gene pairs were required to share at least one CNS. For orthogroup calling, all orthologous genes shared at least one CNS with the reference gene. Gene pairs with a conservation score exceeding 90% of the highest score were classified as paralogues (Supplementary Fig. 4b). A total of 844,525 paralogues was identified across the *Solanum* pan-genome. Sequence evolution pressure rates (*K*_a_/*K*_*s*_) for paralogue pairs were calculated using the R seqinR package (v.4.2-36)^110^. Gene duplication events were classified using DupGen_finder^38^, identifying whole-genome and transposed duplications for gene pairs recognized by both the Conservatory and DupGen_finder tools. Tandem and proximal duplications were defined based on gene positioning: adjacent genes were considered to be tandem duplications, and genes up to ten genes apart were defined as proximal duplications. All other duplicated gene pairs were categorized as dispersed duplications (Supplementary Fig. 4c). Of the identified paralogues, 23,730 were associated with expression groups and were used to compare relationships between sequence evolution pressure rates and protein and *cis*-regulatory divergence across different expression groups. Homologues, orthogroups and paragroups were identified, and the relationships between protein and *cis*-regulatory elements were visualized using custom scripts, which are available at GitHub (https://github.com/pan-sol/pan-sol-pipelines). See Supplementary Table 5 for statistical analysis.

### Statistical analysis

All statistical tests were performed in R. For the quantitative analysis of fruit locule numbers in Figs. 3f and 5c,d and Extended Data Fig. 5b,c, *n* represents the number of fruits quantified. Pairwise comparisons were conducted using Dunnett’s T3 test (R package PMCMRplus v.1.9.10) for multiple comparisons with unequal variances, with the default parameters. Statistical tests and the resulting *P* values are presented in Supplementary Tables 5, 9, 15, 17 and 19.

### Reporting summary

Further information on research design is available in the Nature Portfolio Reporting Summary linked to this article.

## Online content

Any methods, additional references, Nature Portfolio reporting summaries, source data, extended data, supplementary information, acknowledgements, peer review information; details of author contributions and competing interests; and statements of data and code availability are available at 10.1038/s41586-025-08619-6.

## Supplementary information

Supplementary Information Supplementary Results, Supplementary Figs. 1–5 and Supplementary References.

Reporting Summary

Supplementary Tables Supplementary Tables 1–22.

## Data Availability

All data are available within this Article and its Supplementary Information. Raw sequencing data are available at the SRA under BioProject PRJNA1073673. Genome (genome, annotations and variants), expression, VCF files of SVs for the African eggplant pan-genome and phenotypic data, including images of species and accessions, are open access and available at the solpangenomics website (www.solpangenomics.com) and the Solanaceae Genomics Network (SGN; https://solgenomics.net/ftp/genomes/Solanum_pangenomics/). All source data for locule number quantifications are provided in Supplementary Tables 8, 12–14, 16 and 18 and associated summary of statistical tests and analyses are provided in Supplementary Tables 5, 9, 15, 17 and 19. The species distribution maps were generated using the open source osm-liberty package (http://github.com/maputnik/osm-liberty/).

## Code availability

Paralogue expression analysis scripts are available at GitHub (https://github.com/gillislab/pansol_expression_analysis). Other analysis scripts are available at GitHub (https://github.com/pan-sol/pan-sol-pipelines).
