# Supplementary material for: Solanum pan-genetics reveals paralogues as contingencies in crop engineering
Source: Nature. 2025 Mar 5;640(8057):135–45. doi: 10.1038/s41586-025-08619-6 (PMC11964936; doi:10.1038/s41586-025-08619-6)
Supplement: Supplementary file 1 — Supplementary Results, Supplementary Figs. 1–5 and Supplementary References. [file 41586_2025_8619_MOESM1_ESM.pdf]

nature portfolio

https://doi.org/10.1038/s41586-025-08619-6

Supplementary information

Solanum pan-genetics reveals paralogues as contingencies in crop engineering

In the format provided by the authors and unedited

Supplementary Results

for *Solanum* pan-genetics reveals paralogs as contingencies in crop engineering

Pan-genome analysis reveals a complex landscape of gene duplications in *Solanum*.

From the 44,962 total orthogroups identified across the *Solanum* pan-genome, we identified several of them were involved in expansion (26,284) or contraction (37,267) events, with the majority of the evolutionary events occuring at inner nodes involving orthogroup contractions. This indicates that many duplicates likely originated during the WGD events and are shared among species within specific lineages and subclades. While a comprehensive analysis of shared duplications would benefit from broader sampling in our pan-genome, functional enrichment analysis revealed that expanding and contracting orthogroups are predominantly linked to environmental response and secondary metabolism, with species- and clade-specific features potentially associated with shared physiological or environmental adaptations (**Supplementary Fig. 4a, Supplementary Tables 21 and 22**). We then characterized orthogroups based on their representation in the pan-genome, and classified these orthogroups as core (present in 100% of the species), near core (present in >70% of genomes), dispensable (present in 5-70% of species), and private (found in one species only) (**Extended Data Fig. 1b**). For a genus-wide pan-genome, while the total number of private genes continues to grow as more species are added, the number of private genes on a per-species basis decreases as more species from more lineages in the genus were included. Most orthogroups are core (60.6%) or near core (20.2%), while smaller proportions are dispensable (14.3%) or private (0.8%). Finally, 75% of pairs of orthologous genes (designated paragroups) are dispensable or private, suggesting derived paralogs are more genetically flexible than orthologs (**Supplementary Fig. 4b,c**).

Paralogs most frequently originate from WGDs from events many millions of years ago; however, single gene duplications, which typically are more recent and lineage-specific events, collectively dominate the duplication landscape in *Solanum* (**Supplementary Fig. 4d)**. While most of the WGD-derived duplications belong to core orthogroups, single gene duplications show increased bias towards near core and dispensable orthogroups (**Extended Data Fig. 1c**). Analysis of duplication types differentiated according to biological function using a GO

enrichment analysis show that WGD-derived paralog pairs are most strongly associated with dosage-sensitive processes, such as DNA transcription and DNA replication, as well as hormone-mediated signal transduction and response (**Extended Data Fig. 1d**), consistent with previous reports[111,112]. In contrast, and as already shown in many systems[38,113], tandem and proximal duplications are most associated with defense and specialized metabolite biosynthesis, along with diverse functional roles related to environmental responses (**Extended Data Fig. 1d**). Overall, these analyses point to widespread paralog emergence and loss in gene families spanning a multitude of biological functions, which has widespread implications for paralogs shaping genotype-to-phenotype relationships and species-specific contingencies in trait engineering.

Comparison of coding and regulatory sequence evolution across the duplication types.

Paralogous genes functionally diverge through changes in both coding and *cis*-regulatory sequences[114,115]. It remains unclear, however, if the relative contributions of these changes are associated with specific duplication types. To test this, we first used our previously developed algorithm, Conservatory, which simultaneously allows quantification of *cis*-regulatory conservation and improved calling of paralog pairs based on both protein and *cis*-regulatory conservation[107] **(Supplementary Fig. 4b** and **Methods)**. We then incorporated Ka/Ks ratios, as a measure of coding sequence selection, with both protein and *cis*-regulatory conservation to determine relationships in coding and regulatory sequence evolution across the duplication types. This analysis revealed that tandem and proximal duplicates maintain high *cis*-regulatory conservation, while WGD, dispersed, and transposed duplicates show greater retention of *cis*-regulatory conservation as protein sequences diverge, suggesting that expression patterns may remain shared among older, non-local paralogs despite protein divergence **(Supplementary Fig. 4e)**.

Copy number variation in paralogs of flowering time and inflorescence architecture regulators across the *Solanum* pan-genome.

In tomato and many other species, variation in the dosage-sensitive florigen-antiflorigen family members (e.g. the tomato genes *SP*, *SP5G*, *FTL1a*, *FTL1b*, *SP6D*, *SP6A*, *SFT*) enabled selection for accelerated flowering and short stature (determinate) plants, key traits that enabled

growth in northern latitudes, high density planting, and mechanical harvesting[116–118]. We identified numerous copy number variants (CNVs) and loss-of-function mutations affecting orthologs and paralogs of these genes in our pan-genome, suggesting these variants could contribute to variation in flowering time, growth habit, and inflorescence architecture across *Solanum* (**Fig. 3a**). For example, in the genetics of inflorescence architecture, mutations in the MADS-box transcription factor-encoding gene *J2* allowed mechanical harvesting of tomato by eliminating the abscission zone of fruit stems[119,120]. However, co-occurring mutations in its ancestral paralog *EJ2* result in undesirable inflorescence branching[121]. We found one CNV and at least three ancestral losses of *J2* in our pan-genome*,* with most losses occurring in the Eastern Hemisphere Spiny eggplant clade (**Fig. 3a**, **Fig. 1c**). These species may therefore be sensitized for changes in inflorescence branching from natural or engineered *EJ2* mutations, which could quantitatively alter flower production.

African eggplant pan-genomics reveal widespread structural variation and introgressions.

Comparing the African eggplant genomes against the reference showed that, at the sequence level, most of the genome is highly conserved (**Extended Data Fig. 4a**). Over 250,000 structural variants (SVs: defined as variants at least 50 bp in size) were found across all African eggplant samples, mainly towards chromosome ends (**Extended data Fig. 4a,b**). While average SV length was similar across accessions (**Extended Data Fig. 4c**), their density varied between groups, with Gilo possessing the fewest SVs, an expected pattern since the reference African eggplant belongs to the Gilo group (**Extended Data Fig. 4b**). Notably, the SV distribution showed clade-specific SVs and SV clusters shared with the wild ancestor *S. anguivi,* suggesting a history of introgression. We tested this using a window-based Jaccard similarity analysis, which revealed multiple introgressions from *S. anguivi* in the Aculeatum accessions. These introgressions were most conspicuous and extensive on chromosomes 3, 4, 11, and 12 (**Extended Data Fig. 4e,f**). Such widespread introgression suggests recent gene flow from the wild species in the course of African eggplant breeding, and likely explaining the origin of the Aculeatum ornamental types (**Fig. 4b, Extended Data Fig. 4e,f**).

Phenotypes of shoots and fruits from selected *Solanum* species

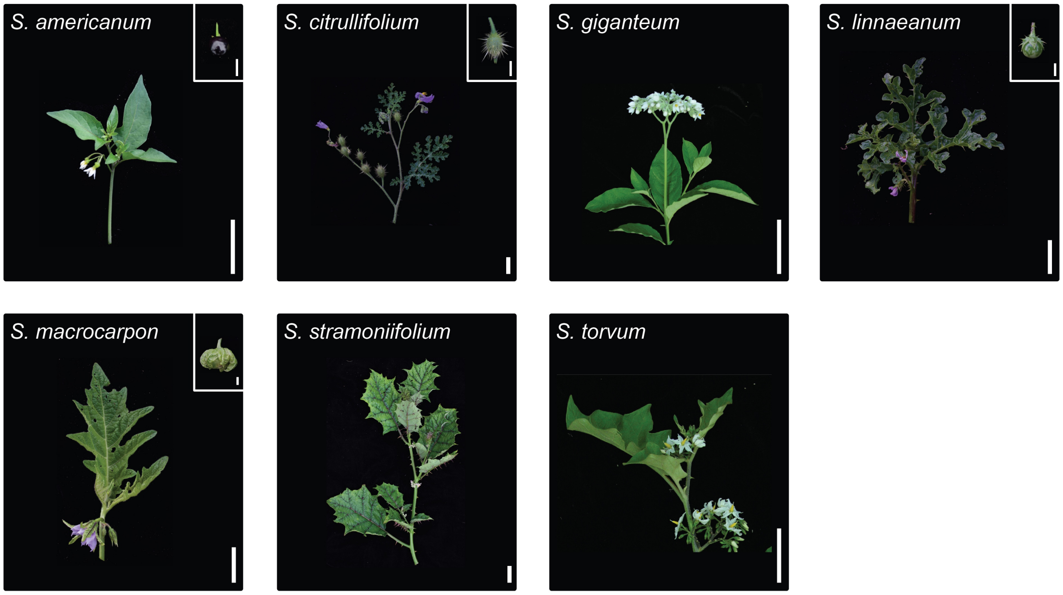

Supplementary Figure 1: Additional images of the *Solanum* pan-genome species. Phenotypic diversity of shoots and fruits (where available) from a subset of the species selected for the *Solanum* pan-genome. Scale bars: 5 cm (shoots) and 1 cm (fruits).

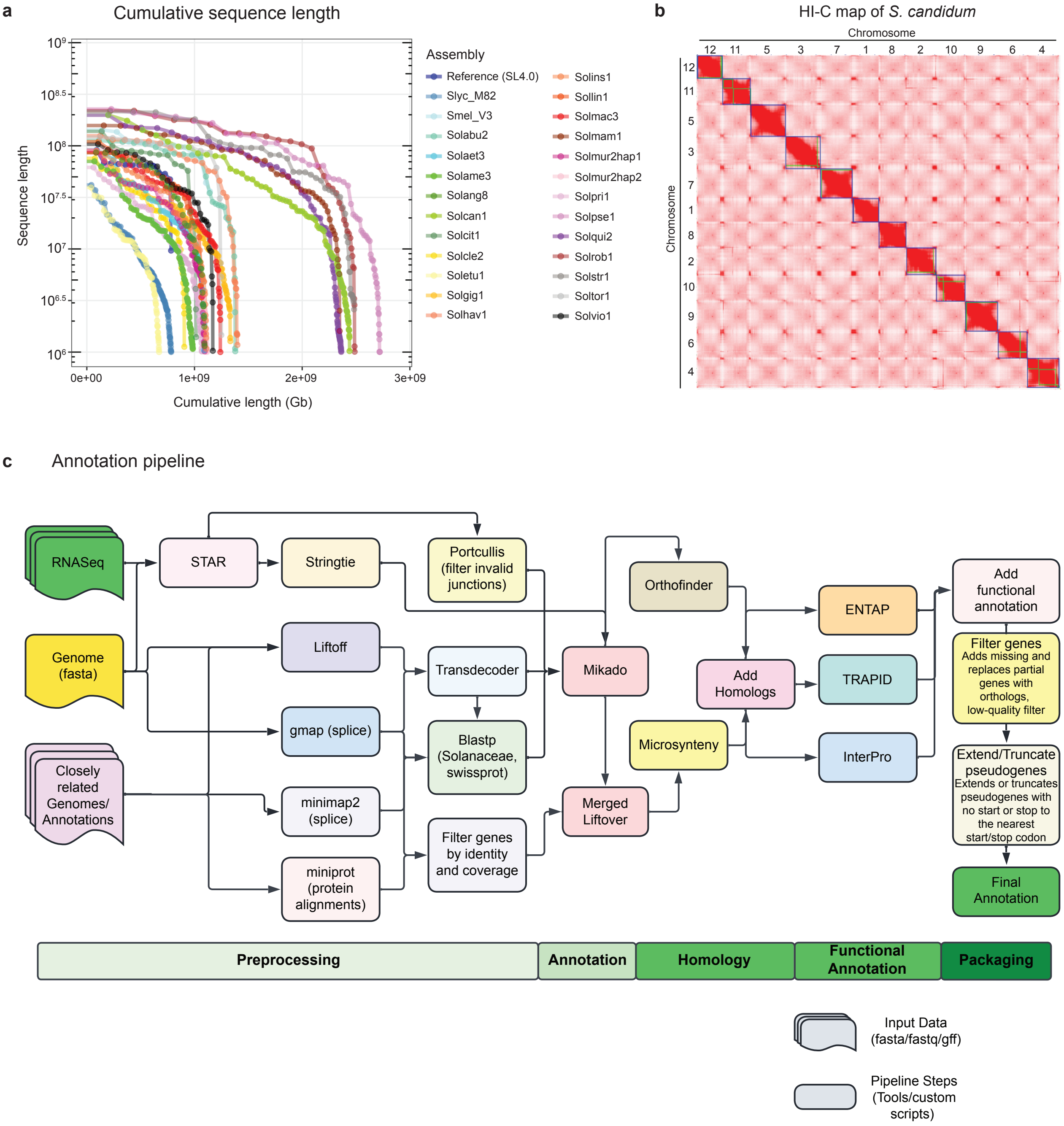

Supplementary Figure 2: Genome assembly features and gene annotation pipeline. (a) Total sizes of the pan-*Solanum* genome assemblies evaluated by cumulative sequence length. Genomes of tomato (*S. lycopersicum*, Heinz SL4.0 and M82) and Brinjal eggplant (*S. melongena*, V3) are shown as references. (b) Hi-C contact map from *S. candidum* shown as a representative example of data used to generate chromosome-scale assemblies. Visualization with Juicebox [122]. (c) Flow chart depicting the gene annotation pipeline used in this study, noting the required input data (RNA-seq data, protein alignments, and genome fasta sequences), tools, and customs scripts. Preprocessing, annotation, homology, functional annotation, and packaging steps are detailed.

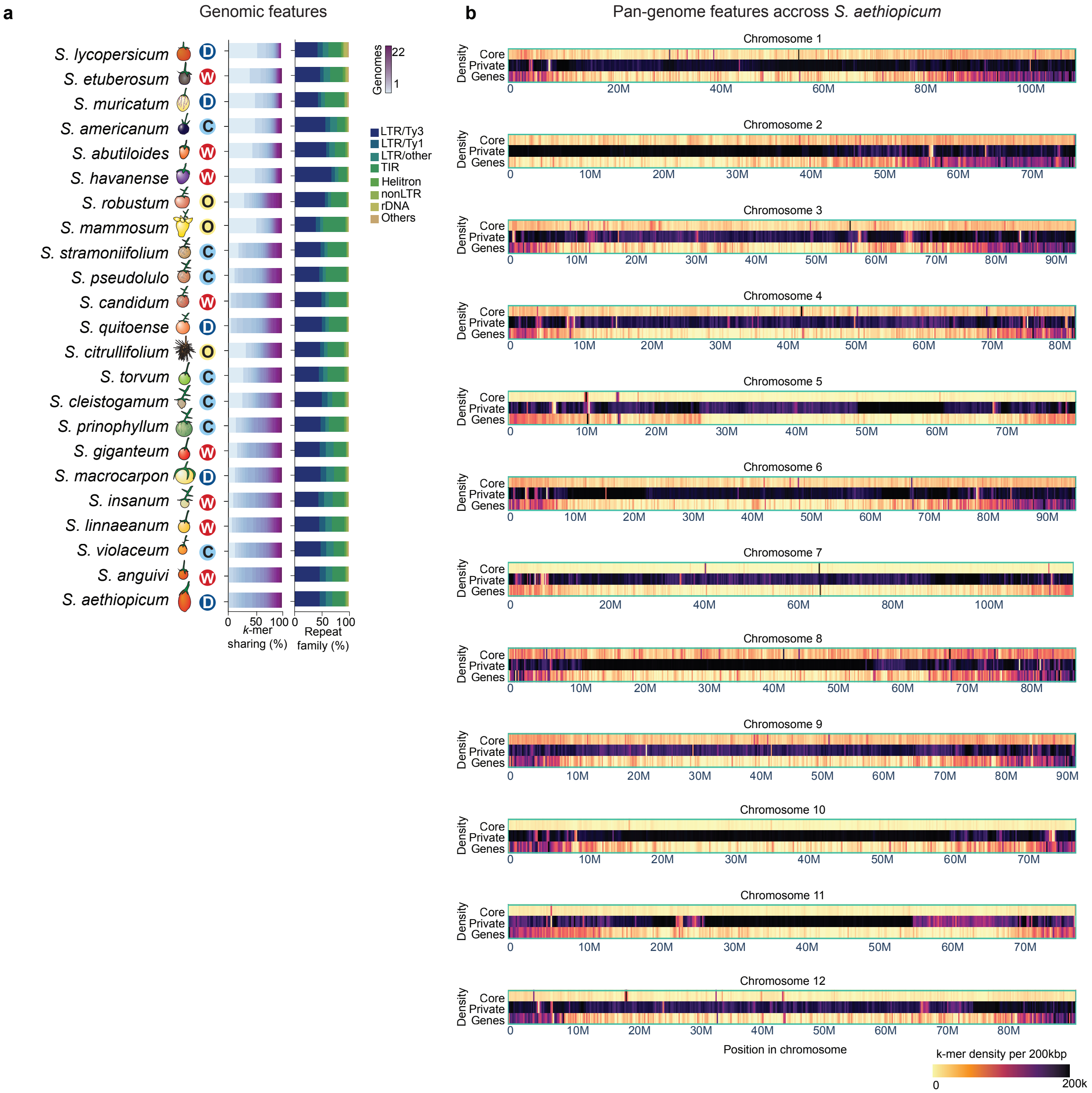

Supplementary Figure 3: *K*-mers and transposable elements features in the *Solanum* pan-genome. (a) Percentage of pan-*k*-mers shared across the pan-genome in each reference (left). Contribution of the different transposable element families in the total repeat landscape of each genome (right). (b) *K*-mer and gene distribution along the 12 chromosomes of *S. aethiopicum*.

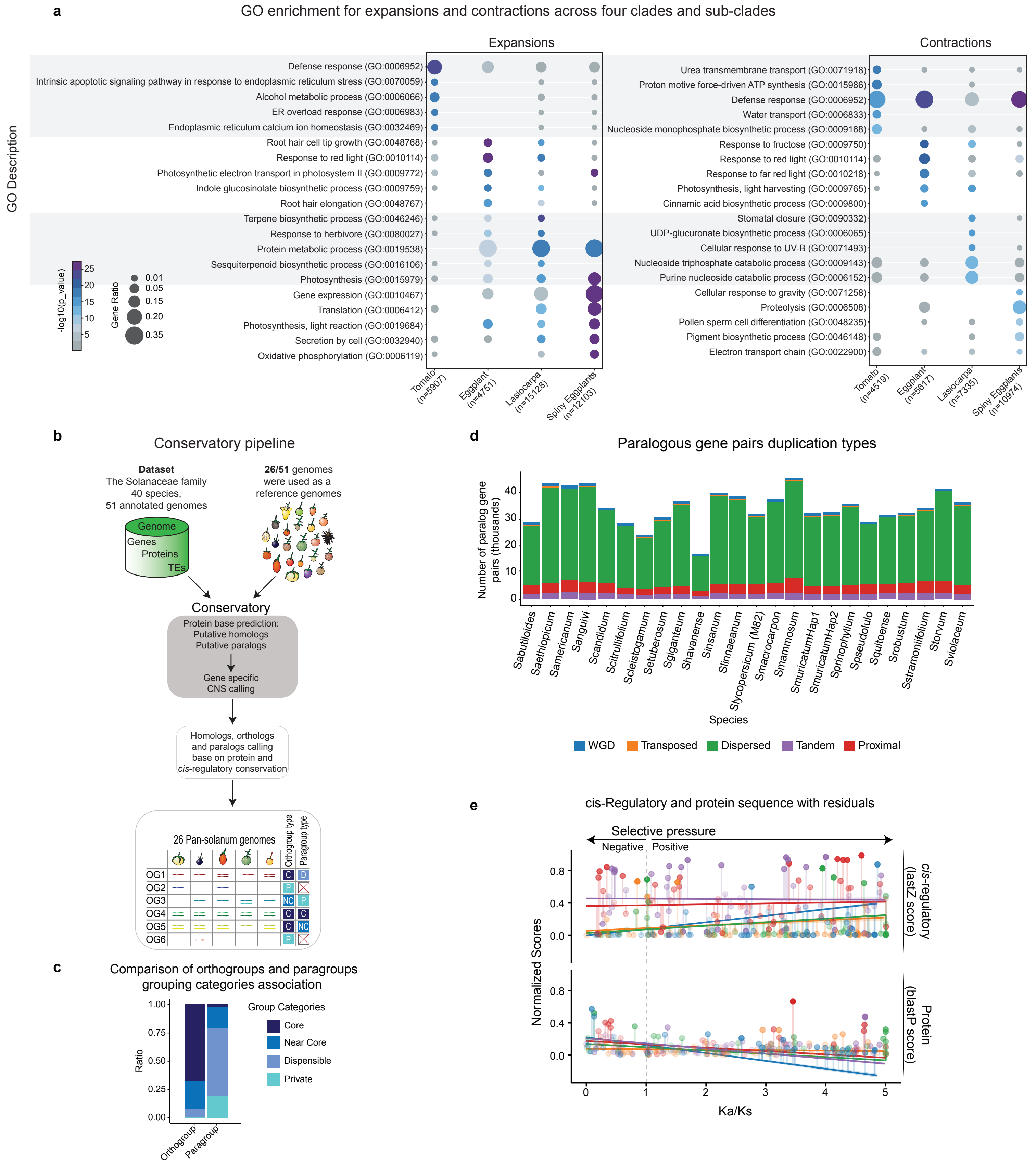

Supplementary Figure 4: Functional enrichment for orthogroup expansions and contractions and Conservatory analysis of paralogous gene pairs across pan-*Solanum* species. (a) Functional enrichment for orthogroup expansions and contractions in tomato, eggplant, and major *Solanum* clades. The top five enriched GO terms per species/clade are shown. Gene ratio represents the number of genes with a specific GO term divided by the total number of genes with GO terms in that category. (b) Flow chart of the Conservatory tool used to define conserved non-coding sequences (CNSs) across pan-genome orthogroups and paragroups. (c) Comparison of orthogroups conservation group size and the subsequent paragroups, defined by the number of species having paralogous genes. Note that ~60% of duplicated gene orthogroups are conserved across all *Solanum* pan-genome species (Core), while less than 1% of the paragroups are Core. (d) Duplicated gene pairs classification of the pan-genome species according to duplication type. (e) Divergence of protein and cis-regulatory sequences across increasing evolutionary pressure, as measured by Ka/Ks values, for the indicated types of gene duplications. For each duplication type the predicted mean, residuals, and 0.95 confidence interval of the normalized BLASTP and LastZ scores are shown (See Supplementary Table 22 for statistical analysis).

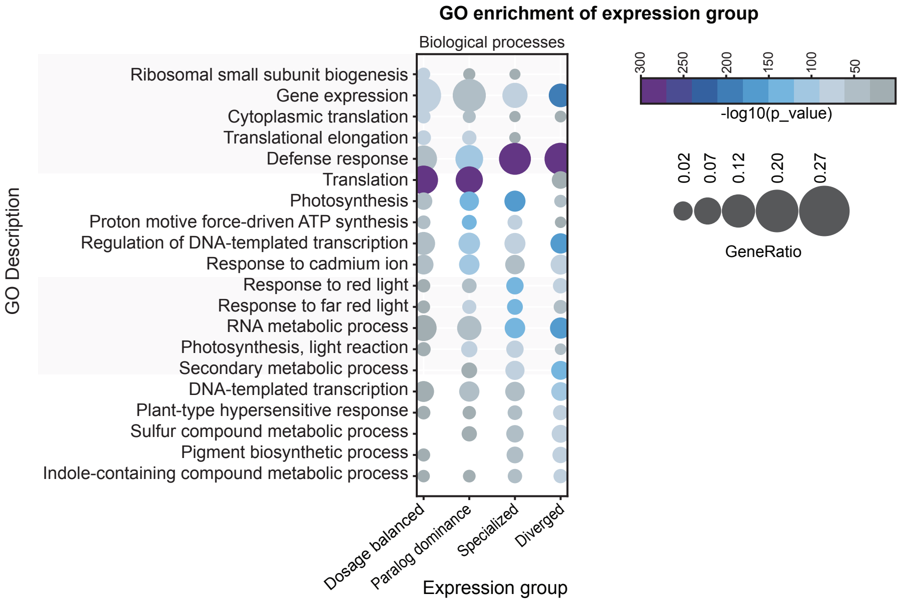

Supplementary Figure 5: Functional enrichment for paralog pairs from the different groups. The top five enriched GO terms per expression group is shown. Circle size represents gene ratio.

References

111. Zhang, T. *et al.* Phylogenomic profiles of whole-genome duplications in Poaceae and landscape of differential duplicate retention and losses among major Poaceae lineages. *Nat. Commun.* **15**, 3305 (2024).

112. Tang, H., Bowers, J. E., Wang, X. & Paterson, A. H. Angiosperm genome comparisons reveal early polyploidy in the monocot lineage. *Proc. Natl. Acad. Sci. U. S. A.* **107**, 472–477 (2010).

113. Qiao, X. *et al.* Different Modes of Gene Duplication Show Divergent Evolutionary Patterns and Contribute Differently to the Expansion of Gene Families Involved in Important Fruit Traits in Pear (Pyrus bretschneideri). *Front. Plant Sci.* **9**, 161 (2018).

114. Baudouin-Gonzalez, L. *et al.* Diverse Cis-Regulatory Mechanisms Contribute to Expression Evolution of Tandem Gene Duplicates. *Mol. Biol. Evol.* **34**, 3132–3147 (2017).

115. Zhong, X., Lundberg, M. & Råberg, L. Divergence in Coding Sequence and Expression of Different Functional Categories of Immune Genes between Two Wild Rodent Species. *Genome Biol. Evol.* **13**, (2021).

116. Nakamichi, N. Adaptation to the local environment by modifications of the photoperiod response in crops. *Plant Cell Physiol.* **56**, 594–604 (2015).

117. Pnueli, L. *et al.* The SELF-PRUNING gene of tomato regulates vegetative to reproductive switching of sympodial meristems and is the ortholog of CEN and TFL1. *Development* **125**, 1979–1989 (1998).

118. Soyk, S. *et al.* Variation in the flowering gene SELF PRUNING 5G promotes day-neutrality and early yield in tomato. *Nat. Genet.* **49**, 162–168 (2017).

119. Budiman, M. A. *et al.* Localization of jointless-2 gene in the centromeric region of tomato chromosome 12 based on high resolution genetic and physical mapping. *Theor. Appl. Genet.* **108**, 190–196 (2004).

120. Rick, C. M. A new jointless gene from the Galapagos L. pimpinellifolium. *TGC Rep* **23**, (1956).

121. Soyk, S. *et al.* Bypassing Negative Epistasis on Yield in Tomato Imposed by a Domestication Gene.

Cell **169**, 1142–1155.e12 (2017).

122. Durand, N. C. *et al.* Juicebox provides a visualization system for Hi-C contact maps with unlimited zoom. *Cell Syst.* **3**, 99–101 (2016).
