# Supplementary material for: Solanum pan-genetics reveals paralogues as contingencies in crop engineering
Source: Nature. 2025 Mar 5;640(8057):135–45. doi: 10.1038/s41586-025-08619-6 (PMC11964936; doi:10.1038/s41586-025-08619-6)
Supplement: Supplementary file 2 — Reporting Summary [file 41586_2025_8619_MOESM2_ESM.pdf]

nature portfolio

Corresponding author(s): J. Gillis, J. Van Eck, M. C. Schatz, Z. B. Lippman

Last updated by author(s): January 8th, 2025

Reporting Summary

Nature Portfolio wishes to improve the reproducibility of the work that we publish. This form provides structure for consistency and transparency in reporting. For further information on Nature Portfolio policies, see our Editorial Policies and the Editorial Policy Checklist.

Statistics

For all statistical analyses, confirm that the following items are present in the figure legend, table legend, main text, or Methods section.

n/a	Confirmed	
☐	☒	The exact sample size (*n*) for each experimental group/condition, given as a discrete number and unit of measurement
☐	☒	A statement on whether measurements were taken from distinct samples or whether the same sample was measured repeatedly
☐	☒	The statistical test(s) used AND whether they are one- or two-sided<br>*Only common tests should be described solely by name; describe more complex techniques in the Methods section.*
☒	☐	A description of all covariates tested
☐	☒	A description of any assumptions or corrections, such as tests of normality and adjustment for multiple comparisons
☐	☒	A full description of the statistical parameters including central tendency (e.g. means) or other basic estimates (e.g. regression coefficient) AND variation (e.g. standard deviation) or associated estimates of uncertainty (e.g. confidence intervals)
☐	☒	For null hypothesis testing, the test statistic (e.g. *F*, *t*, *r*) with confidence intervals, effect sizes, degrees of freedom and *P* value noted<br>*Give P values as exact values whenever suitable.*
☒	☐	For Bayesian analysis, information on the choice of priors and Markov chain Monte Carlo settings
☒	☐	For hierarchical and complex designs, identification of the appropriate level for tests and full reporting of outcomes
☐	☒	Estimates of effect sizes (e.g. Cohen's *d*, Pearson's *r*), indicating how they were calculated

Our web collection on statistics for biologists contains articles on many of the points above.

Software and code

Policy information about availability of computer code

Data collection	Sequencing data were recorded as fastq files (see Methods), quantitative phenotypic data was collected manually in fields and greenhouses and recorded in Microsoft Excel (v18.85) (see Methods).
Data analysis	KMC3 (v3.2.1), GenomeScope (v2.0), Hifiasm (exact parameters and software version varied between samples based on the level of estimated heterozygosity and are reported in Supplementary Table 2), Bionano Solve Hybrid Scaffold (v3 8.2, default parameters), Juicer (v0.7.17-r1198-dirty), Juicebox (v1.11.08), RagTag scaffold (v2.1.0, default parameters), Merqury (V1.3), FastQC (v0.11.9), trimmomatic (v0.39), STAR (v2.7.5c), Stringtie2 (v2.1.2), Portcullis (v1.2.0), Liftoff (v1.6.3), Gmap (version 2020-10-14), Minimap2 (v2.17-r941), Mikado (v2.Orc2), Microsynteny and Orthofinder (v2.5.2), Miniprot2 (v2.28), ENTAP (v0.10.8), TEsorter (v1.4.7), BUSCO (v5.7.0), MAFFT (v7), trimAl (v1.5.0), IQ-TREE2 (v2), ASTRAL-III (v5.7.3), Newick Utilities (v1.5.0), R packages: ggtree (v3.19), treeio (v3.19), ggplot2 (v3.5.0), seqinR (v4.2-36), PMCMRplus (v1.9.10), CAFE5 (v1.1), GOATOOLS (v1.4.12), DupGen_finder, Exonerate (V2.2.0), Clinker (v0.0.29), KaKs _Calculator (v2.0), QTL-Seq software package (v2.2.4), GENESPACE (v1.3.1), EDTA (v2.1.5), panEDTA, LAI (b3.2), Conservatory (v2.0), ImageLab Software (v6.1, default parameters), OSM Liberty.<br><br>Code availability: Paralog expression analysis scripts are available at github.com/gillislab/pansol_expression_analysis. Other analysis scripts are available within github.com/pan-sol/pan-sol-pipelines.

For manuscripts utilizing custom algorithms or software that are central to the research but not yet described in published literature, software must be made available to editors and reviewers. We strongly encourage code deposition in a community repository (e.g. GitHub). See the Nature Portfolio guidelines for submitting code & software for further information.

Data

Policy information about availability of data

All manuscripts must include a data availability statement. This statement should provide the following information, where applicable:

- Accession codes, unique identifiers, or web links for publicly available datasets
- A description of any restrictions on data availability
- For clinical datasets or third party data, please ensure that the statement adheres to our policy

All data are available within this Article and its Supplementary Information. Raw sequencing data are available in the SRA under BioProject PRJNA1073673. Genome (genome, annotations, variants), expression, VCF files of SVs for the African eggplant pan-genome, and phenotypic data, including images of species and accessions, are open access and available at the "solpangenomics" website (www.solpangenomics.com) and the Solanaceae Genomics Network (SGN: https://solgenomics.net/ftp/genomes/Solanum_pangenomics/). All source data for locule number quantifications are found in Supplementary Tables 8, 12-14, 16, and 18 and associated summary of statistical tests and analyses are found in Supplementary Tables 9, 15, 17, and 19.

Additional databases used in this study are: Uniprot/Swissprot, TREMBL, RefSeq, Solanaceae proteins, TIGRFAM, Gene Ontology, PLAZA dicots (v5.0), InterProScan 5 with Pfam, TRAPID.

Research involving human participants, their data, or biological material

Policy information about studies with human participants or human data. See also policy information about sex, gender (identity/presentation), and sexual orientation and race, ethnicity and racism.

Reporting on sex and gender	NA
Reporting on race, ethnicity, or other socially relevant groupings	NA
Population characteristics	NA
Recruitment	NA
Ethics oversight	NA

Note that full information on the approval of the study protocol must also be provided in the manuscript.

Field-specific reporting

Please select the one below that is the best fit for your research. If you are not sure, read the appropriate sections before making your selection.

☒ Life sciences ☐ Behavioural & social sciences ☐ Ecological, evolutionary & environmental sciences

For a reference copy of the document with all sections, see nature.com/documents/nr-reporting-summary-flat.pdf

Life sciences study design

All studies must disclose on these points even when the disclosure is negative.

Sample size	Sample size for RNA-Seq was based on four independent samples, as done previously (Alonge, Wang et al., 2020). No statistic methods were used to predetermine sample size for quantitative phenotypic analyses. Required experimental sample size was estimated based on our past experience performing similar experiments including greenhouse and field tests (see for example Kwon et al., 2022, Alonge, Wang et al., 2020, Rodríguez-Leal et al., 2017).
Data exclusions	Mechanically damaged and diseased plants were excluded from the analyses to minimize environmental effects and focus on the genetic control of the observed developmental phenotypes.
Replication	All relevant information is presented in figure legends, methods, and supplementary data files. Individual replicates (e. g. tissue samples, plants, shoots, flowers and fruits) are indicated and at least four independent replicates were analyzed for each experiment. Raw phenotypic data are provided in supplementary data files.
Randomization	For the QTL-sequencing experiments, two independent segregating F2 mapping populations of 144 and 135 individual plants, respectively, were randomly sown and transplanted in an agricultural field. These randomized and blinded experiments allowed the identification of the locule number modifiers in African eggplant, which were confirmed by genotyping.
Blinding	For the QTL-sequencing experiments, two independent segregating F2 mapping populations of 144 and 135 individual plants, respectively, were randomly sown and transplanted in an agricultural field. These randomized and blinded experiments allowed the identification of the locule number modifiers in African eggplant, which were confirmed by genotyping.

Reporting for specific materials, systems and methods

We require information from authors about some types of materials, experimental systems and methods used in many studies. Here, indicate whether each material, system or method listed is relevant to your study. If you are not sure if a list item applies to your research, read the appropriate section before selecting a response.

Materials & experimental systems

n/a	Involved in the study	
☒	☐	Antibodies
☒	☐	Eukaryotic cell lines
☒	☐	Palaeontology and archaeology
☒	☐	Animals and other organisms
☒	☐	Clinical data
☒	☐	Dual use research of concern
☐	☒	Plants

Methods

n/a	Involved in the study	
☒	☐	ChIP-seq
☒	☐	Flow cytometry
☒	☐	MRI-based neuroimaging

Dual use research of concern

Policy information about dual use research of concern

Hazards

Could the accidental, deliberate or reckless misuse of agents or technologies generated in the work, or the application of information presented in the manuscript, pose a threat to:

No	Yes	
☒	☐	Public health
☒	☐	National security
☒	☐	Crops and/or livestock
☒	☐	Ecosystems
☒	☐	Any other significant area

Experiments of concern

Does the work involve any of these experiments of concern:

No	Yes	
☒	☐	Demonstrate how to render a vaccine ineffective
☒	☐	Confer resistance to therapeutically useful antibiotics or antiviral agents
☒	☐	Enhance the virulence of a pathogen or render a nonpathogen virulent
☒	☐	Increase transmissibility of a pathogen
☒	☐	Alter the host range of a pathogen
☒	☐	Enable evasion of diagnostic/detection modalities
☒	☐	Enable the weaponization of a biological agent or toxin
☒	☐	Any other potentially harmful combination of experiments and agents

Plants

Seed stocks	Species name, accession number, and seed stock source for all seed material used in this study are provided in Supplementary Tables 1 and 10.
Novel plant genotypes	Novel plant genotypes were obtained in this study. Novel CLV3 mutant alleles were generated by gene editing for S. cleistogamum, S. prinophyllum, and S. aethiopicum. Novel SCPL25 mutant alleles were generated for S. lycopersicum.
Authentication	Species and cultivar authentication was achieved by expert opinion on Solanum species from E. B. Kizito, S. Knaap, T. E. Särkinen, and F. Roda.
